# Salivary Gland Transcriptomes and Proteomes of *Phlebotomus tobbi* and *Phlebotomus sergenti*, Vectors of Leishmaniasis

**DOI:** 10.1371/journal.pntd.0001660

**Published:** 2012-05-22

**Authors:** Iva Rohoušová, Sreenath Subrahmanyam, Věra Volfová, Jianbing Mu, Petr Volf, Jesus G. Valenzuela, Ryan C. Jochim

**Affiliations:** 1 Department of Parasitology, Faculty of Science, Charles University in Prague, Prague, Czech Republic; 2 Vector Molecular Biology Section, Laboratory of Malaria and Vector Research, National Institute of Allergy and Infectious Diseases, National Institutes of Health, Rockville, Maryland, United States of America; 3 Malaria Genomics Section, Laboratory of Malaria and Vector Research, National Institute of Allergy and Infectious Diseases, National Institutes of Health, Rockville, Maryland, United States of America; National Yang-Ming University, Taiwan

## Abstract

**Background:**

*Phlebotomus tobbi* is a vector of *Leishmania infantum*, and *P. sergenti* is a vector of *Leishmania tropica*. *Le. infantum* and *Le. tropica* typically cause visceral or cutaneous leishmaniasis, respectively, but *Le. infantum* strains transmitted by *P. tobbi* can cause cutaneous disease. To better understand the components and possible implications of sand fly saliva in leishmaniasis, the transcriptomes of the salivary glands (SGs) of these two sand fly species were sequenced, characterized and compared.

**Methodology/Principal Findings:**

cDNA libraries of *P. tobbi* and *P. sergenti* female SGs were constructed, sequenced, and analyzed. Clones (1,152) were randomly picked from each library, producing 1,142 high-quality sequences from *P. tobbi* and 1,090 from *P. sergenti*. The most abundant, secreted putative proteins were categorized as antigen 5-related proteins, apyrases, hyaluronidases, D7-related and PpSP15-like proteins, ParSP25-like proteins, PpSP32-like proteins, yellow-related proteins, the 33-kDa salivary proteins, and the 41.9-kDa superfamily of proteins. Phylogenetic analyses and multiple sequence alignments of putative proteins were used to elucidate molecular evolution and describe conserved domains, active sites, and catalytic residues. Proteomic analyses of *P. tobbi* and *P. sergenti* SGs were used to confirm the identification of 35 full-length sequences (18 in *P. tobbi* and 17 in *P. sergenti*). To bridge transcriptomics with biology *P. tobbi* antigens, glycoproteins, and hyaluronidase activity was characterized.

**Conclusions:**

This analysis of *P. sergenti* is the first description of the subgenus *Paraphlebotomus* salivary components. The investigation of the subgenus *Larroussius* sand fly *P. tobbi* expands the repertoire of salivary proteins in vectors of *Le. infantum*. Although *P. tobbi* transmits a cutaneous form of leishmaniasis, its salivary proteins are most similar to other *Larroussius* subgenus species transmitting visceral leishmaniasis. These transcriptomic and proteomic analyses provide a better understanding of sand fly salivary proteins across species and subgenera that will be vital in vector-pathogen and vector-host research.

## Author Summary

Phlebotomine female sand flies require a blood meal for egg development, and it is during the blood feeding that pathogens can be transmitted to a host. *Leishmania* parasites are among these pathogens and can cause disfiguring cutaneous or even possibly fatal visceral disease. The *Leishmania* parasites are deposited into the bite wound along with the sand fly saliva. The components of the saliva have many pharmacologic and immune functions important in blood feeding and disease establishment. In this article, the authors identify and investigate the protein components of saliva of two important vectors of leishmaniasis, *Phlebotomus tobbi* and *P. sergenti*, by sequencing the transcriptomes of the salivary glands. We then compared the predicted protein sequences of these salivary proteins to those of other bloodsucking insects to elucidate the similarity in composition, structure, and enzymatic activity. Finally, this descriptive analysis of *P. tobbi* and *P. sergenti* transcriptomes can aid future research in identifying molecules for epidemiologic assays and in investigating sand fly-host interactions.

## Introduction

Sand flies are bloodsucking nematoceran Diptera that transmit the protozoan parasites of the genus *Leishmania*. Similar to that of other bloodsucking arthropods, sand fly saliva comprises antihemostatic, immunomodulatory, and antigenic components. The saliva is deposited into the host skin every time the sand fly ingests a blood meal to facilitate feeding. Also during the bite by an infected sand fly, *Leishmania* parasites are egested into the wound with the saliva. Sand fly saliva can enhance *Leishmania* infection in naive mice [1], [2]. Conversely, pre-exposure of mice to sand fly saliva conferred a protective effect against *Leishmania* infection [3], [4]. Even single salivary proteins have been characterized as potential *Leishmania* vaccine candidates in mouse, hamster, and dog models of cutaneous or visceral leishmaniasis [5]–[9].

The potent effects of sand fly saliva stimulate a protective host cellular immune response [3]–[9], and the antigenic nature of saliva also provides a humoral immunity measurement of host exposure to sand fly bites already used in several human epidemiological studies [10]–[18]. Identifying markers of vector exposure based on anti-saliva antibodies are essential in epidemiologic and vector control surveillance [15], [16], [18], [19]–[21]. However, anti-saliva antibodies are highly specific [12], [16], [22] and with over 80 species of sand flies implicated in *Leishmania* transmission, it is vital to continue describing the salivary proteins in the search for markers of exposure as well as vaccine candidates.

Sand fly salivary gland proteins have been well studied in *Lutzomyia longipalpis* [23], [24] and *Phlebotomus papatasi* [6]. Recently, transcriptomic and proteomic data have been published for several other sand fly species, vectors of visceral (*P. ariasi*, *P. argentipes*, and *P. perniciosus*) and cutaneous (*P. arabicus*, *P. duboscqi*) forms of leishmaniasis [25]–[28].

To broaden the repertoire of subgenus *Larroussius* salivary proteins and provide the first report from a subgenus *Paraphlebotomus* sand fly, we prepared and analyzed the transcriptomes and proteomes of *P. tobbi* and *P. sergenti*, both proven vectors in the Old World. *Phlebotomus sergenti*, subgenus *Paraphlebotomus*, is the main vector of *Le. tropica*, principally an agent of cutaneous leishmaniasis [29]–[31]. *Phlebotomus tobbi*, on the other hand, is an important vector of *Le. infantum* [32] together with the taxonomically related *P. ariasi* and *P. perniciosus*, sand flies of the subgenus *Larroussius* [29], [33], [34]. In contrast to other members of the subgenus, *P. tobbi* transmits the cutaneous form of the *Le. infantum* [32]. Additionaly, we characterized *P. tobbi* antigens, glycoproteins, and hyaluronidase activity; the later one compared with 6 sand fly species belonging to vectors of cutaneous or visceral leishmaniases.

## Methods

### Sand fly salivary glands

Colonies of *P. tobbi* (originating from Turkey), *P. papatasi* (Turkey), *P. sergenti* (Israel), *P. argentipes* (India), *P. arabicus* (Israel), *P. perniciosus* (Spain), and *L. longipalpis* (Brazil) were kept in the insectary of Charles University in Prague as described in [35]. The *P. sergenti* colony, originating from Turkey, was reared in similar conditions at the Laboratory of Malaria and Vector Research, National Institutes of Health (Rockville, MD, USA). For mRNA extraction, salivary glands (SGs) from non-bloodfed 1- to 2-day-old female sand flies were dissected and stored in RNA Later (Ambion, Inc., Austin, TX, USA). For other assays and analysis, SGs of non-bloodfed 5- to 7-day-old females were stored at −70°C; SGs were stored in NuPAGE LDS sample buffer (Invitrogen, Carlsbad, CA, USA) for proteome analysis and in Tris buffer (20 mM Tris, 150 mM NaCl, pH 7.8) for hyaluronidase assays, affinity blot, and immunoblot. Before use, samples were homogenized by three freeze-thaw cycles in liquid nitrogen. Protein concentration in resulting SG homogenate (SGH) was measured on Qubit Fluorometer (Invitrogen) following manufacturer's guidelines.

### Salivary gland cDNA library construction and sequencing

An SG cDNA library was constructed from *P. sergenti* (Turkey) and *P. tobbi*. MicroFastTrack mRNA isolation kit (Invitrogen) was used to isolate SG mRNA from 40 SG pairs dissected into 20 µl of RNA Later (Ambion). A cDNA library was constructed using SMART™ cDNA Library Construction Kit (BD Clontech, Palo Alto, CA, USA) following the manufacturer's protocol, with some modifications as described in [36]. For each species, three cDNA libraries were constructed according to PCR product size – large, medium, and small. PCR amplicons were washed and concentrated to 4–7 µl on Microcon YM-100 columns (Millipore, Billerica, MA, USA). Concentrated samples (3 µl) were ligated into the λTripleEx2 vector and packed into the phage particles with Gigapack III Gold Packaging Extract (Stratagene, La Jolla, CA, USA). Phage libraries were used to infect the log-phase XL-1 Blue *Escherichia coli* (Clontech) plated onto four LB agar plates per each library size. Transfected plaques were randomly selected and transferred into 96-well V-shape plates with 75 µl of ultrapure water per well. Four 96-well plates of phage were picked per each library size, resulting in 12 plates (1,152 clones) per sand fly species. Phages (3 µl) were subjected to PCR using FastStart PCR Master Mix (Roche, Molecular Biochemicals, Indianopolis, IN, USA) and vector-specific primers (PT2F1 5′-AAGTACTCTAGCAATTGTGAGC-3′ and PT2R1 5′-CTCTTCGCTATTACGCCAGCTG-3′). Amplification conditions were as follows: 1 hold of 75°C for 3 min, 1 hold of 94°C for 2 min, 34 cycles of 94°C for 1 min, 49°C for 1 min, and 72°C for 2 min. The final elongation step lasted for 10 min at 72°C. Products were cleaned using ExcelaPure 96-well UF PCR Purification Plates (Edge Biosystems, Gaithersburg, MD, USA) and cleaned PCR products were used as a template for cycle-sequencing reaction using BigDye Terminator v3.1 cycle sequencing kit (Applied Biosystems, Fullerton, CA, USA) and a vector-specific forward primer (PT2F3 5′-CTCGGGAAGCGCGCCATTGT-3′). Products of cycle-sequencing reaction were cleaned using Sephadex and MultiScreen HV Plates (Millipore), dried, resuspended in formamide, and stored at −20°C until sequenced on an ABI 3730XL 96-Capillary DNA Sequencer (Applied Biosystems).

### Proteome analysis

For mass spectrometric (MS) analysis, SGH samples of *P. sergenti* (Turkey) and *P. tobbi* were dissolved in Laemmli sample buffer in parallel with or without 2-mercaptoethanol and electrophoretically separated on 12% polyacrylamide SDS minigel with initial voltage 80 V and 120 V upon entry of sample to the gel. Gels were stained for total proteins with Coomassie Brilliant Blue R-250. Individual bands were cut, destained and digested as was described in [37]. Samples (0.5 µl) were transferred to a 384 spot stainless steel MALDI target (AB Sciex, Framingham, MA, USA) and let to dry. Dried droplets were covered with a 0.5 µl drop of alpha-cyano-hydroxycinnamic acid (Fluka, Switzerland) solution (2 mg/ml in 80% acetonitrile) and allowed to dry. Spectra were acquired with 4800 Plus MALDI TOF/TOF analyzer (AB Sciex) equipped with a Nd∶YAG laser (355 nm; firing rate 200 Hz). Voltages were set as follows: source1 20 kV, grid1 16 kV, source1 lens 10 kV, lens1 5 kV, mirror1 14.085 kV, mirror2 20.3 kV and reflector detector 1.905 kV. Digitizer bin size was set to 0.5 ns, vertical scale 0.5 V, vertical offset 0.0, input bandwith 500 MHz. Spectra were externally calibrated using ProteoMass peptide MALDI calibration kit (Sigma-Aldrich). Spectra were recorded in the range 700 to 4000 Da, focus mass 2100 Da. Spectra were summed from 40 positions per 50 shots, 2000 shots in total. Spectra were processed by 4000 Series Explorer version 3.5.3 (AB Sciex) without smoothing; baseline subtraction was performed with peak width set to 50. Spectra were deisotoped and peaks with a local signal-to-noise ratio greater than 5 were picked and searched by local Mascot v. 2.1 (Matrix Science, Boston, MA, USA) against a database of protein sequences derived from the cDNA library. Database search criteria were as follows: enzyme: trypsin; taxonomy: none; fixed modification: carbamidomethylation; variable modification: methionine oxidation; peptide mass tolerance: 80 ppm; one missed cleavage allowed. Only hits that were scored as significant (P<0.05) were included.

The data associated with this manuscript may be downloaded from ProteomeCommons.org Tranche using the following hash: mCZfFsOLaBtSfR+Jh6o8OwEgjrqDp4m3VntpJdAPqPGFNzNpTPry8IhEuGeLw9

TmpHcTRMSiiuiNNRL/6xP65TLvyNwAAAAAAAADGQ =   = . The hash may be used to prove exactly what files were published as part of this manuscript's data set, and the hash may also be used to check that the data has not changed since publication.

### Bioinformatic and phylogenetic analysis

Expression sequence tags (ESTs) were analyzed using the dCAS software (Desktop cDNA Annotation System, version 1.4.3) [38] with all third-party components recommended: CAP3 assembler program [39], Phred [40], [41], and BLAST programs [42]. Sequences with Phred quality scores lower than 25 were removed, as well as primers and vector sequences. Resulting sequences were grouped based on nucleotide homology of 90% identity over 100 residues and aligned into consensus transcript sequences (contigs) using the CAP3 sequence assembly program. BLAST programs were used to compare contigs and singletons (contigs with a single sequence) to the non-redundant protein database of the NCBI, the Gene Ontology database (GO) [43], to COG conserved domains database [44], Protein Family database (Pfam) [45], Simple Modular Architecture Tool database (SMART) [46], and to rRNA Nucleotide Sequences, and Mitochondrial and Plasmid Sequence (MIT-PLA) databases available from NCBI. The three frame translations of each dataset were submitted to the SignalP server [47] to detect signal peptides. The grouped and assembled sequences, BLAST results, and SignalP results combined by dCAS software in an Excel spreadsheet were manually verified and annotated. Additionally, glycosylation sites were determined in selected sequences using NetNGlyc prediction server [48]. For phylogenetic analysis, protein sequences without signal peptide were aligned using ClustalX (version 2.0) [49] with related sequences obtained from GenBank and manually refined in BioEdit 7.0 editing software. For each alignment, best substitution matrix was determined by ProtTest software, version 2.0 [50]. This matrix was then used by TREE-PUZZLE 5.2 [51] to reconstruct maximum likelihood phylogenetic trees from the protein alignments using quartet puzzling with 1000 puzzling steps. Resulting trees were visualized in MEGA 4 [52]. All protein and nucleotide accession numbers mentioned in the text, tables and figures are listed in Text S1.

### Hyaluronidase activity

Hyaluronidase activity was compared between seven sand fly species: *P. tobbi*, *P. sergenti* (Israel), *P. papatasi*, *P. argentipes*, *P. arabicus*, *P. perniciosus*, and *L. longipalpis*. Hyaluronidase activity in SGs was quantified using a sensitive assay on microtitration plates coupled with biotinylated HA (bHA). bHA, prepared as described in [53], was immobilized onto Covalink NH microtiter plates (Nunc, Placerville, NJ, USA) using the method in [54] at a final concentration of 1 µg bHA per well. The plates were incubated overnight at 4°C and washed three times in PBS (containing 2 M NaCl and 50 mM MgSO_4_, pH 7.2). Plates with immobilized bHA were blocked with 1% BSA in PBS for 45 min, washed and equilibrated to pH 5.0 (0.1 M acetate, 0.1 M NaCl, 0.1% Triton X-100, pH 5.0), the pH optimum for sand fly salivary hyaluronidase [53]. SGHs were incubated for 45 min at 37°C in triplicate at a final concentration of 0.5 gland per well. As a standard, bovine hyaluronidase (Sigma) at a concentration of 0.01 Turbidity Reducing Units (TRU)/µl was serially diluted in 0.1 M acetate buffer (0.1 M NaCl, 0.1% Triton X-100, pH 4.5). Wells without bHA or enzyme were used as controls. The reaction was terminated by 6 M guanidine 200 µl/well. Plates were washed in PBS (containing 2 M NaCl, 50 mM MgSO_4_, 0.05% Tween 20, pH 7.2) and then equilibrated with PBS, 0.1% Tween 20, pH 7.2. Avidin-peroxidase (Sigma) was added at a final concentration of 0.2 µg/well and incubated for 30 min at room temperature. Color reaction was developed with o-phenylenediamine substrate in 0.1 M citrate-phosphate buffer (pH 5.5). After 10 min in dark, plates were read at 492 nm (Tecan-Infinite M 200 Fluorometer; Schoeller Instruments, Prague, Czech Republic). The obtained results were expressed as relative TRU (rTRU). Three independent experiments were performed with a different set of SGH samples in each experiment.

For hyaluronidase zymography, 8% polyacrylamide gels (0.75 mm thick) were copolymerized with 0.002% hyaluronic acid (HA). As the hyaluronidase activities and band patterns varied among sand fly species, different loads were used per lane to obtain bands of equal intensity. The equivalent of 1/2 gland (*L. longipalpis* and *P. sergenti*) or 1/20 gland (other tested species) was loaded for zymography under non-reducing conditions, and the equivalent of 2.5 glands (*L. longipalpis* and *P. sergenti*) or 1/4 gland (other tested species) was loaded for zymography under reducing conditions. The total protein content per lane was as follows (non-reducing/reducing conditions): *L. longipalpis* = 110/550 ng; *P. papatasi* = 12.5/62.5 ng; *P. sergenti* = 140/700 ng; *P. argentipes* = 14/70 ng; *P. arabicus* = 10.5/52.5 ng; *P. tobbi* = 10/50 ng; *P. perniciosus* = 10.5/52.5 ng. For reducing conditions, samples were treated with 3% 2-mercaptoethanol for 40 min at 45°C. SDS-PAGE electrophoresis was carried out using Mini-Protean II apparatus (Bio-Rad, Hercules, CA, USA) and constant voltage at 150 V. After electrophoresis, gels were rinsed 2×20 min in 0.1 M Tris, pH 7.8, and 20 min in 0.1 M acetate buffer, pH 5.5 (both with 1% Triton X-100 to wash out SDS) and then incubated in 0.1 M acetate buffer (without detergent) for 120 min at 37°C. The gels were then washed in water, soaked in 50% formamide for 30 min and stained in Stains-all (Sigma, St. Louis, MO, USA) solution (100 mg/ml in 50% formamide) for 24 h in the dark. Hyaluronidase activity was visible as a pink band on a dark blue background.

### Immunoblotting

Immunoblot was performed using *P. tobbi* SGH separated by SDS-PAGE on 10% polyacrylamide gel under non-reducing conditions using the Mini-Protean III apparatus (Bio-Rad). Separated proteins were electrotransferred onto nitrocellulose (NC) membrane by iBlot Dry Blotting System (Invitrogen). After transfer, the NC membrane was cut into strips with the equivalent of four glands/strip and free binding sites were blocked by 5% low-fat dried milk in 20 mM Tris buffer with 0.05% Tween (Tris-Tw) overnight at 4°C. The strips were then incubated with serum obtained from rabbit repeatedly exposed to *P. tobbi* females. Serum was diluted 1∶250 in Tris-Tw and incubated with *P. tobbi* proteins for 1 h, followed by 1 h incubation with peroxidase-conjugated swine anti-rabbit IgG (Sevapharma, Prague, Czech Republic) diluted 1∶1,000 in Tris-Tw. Substrate solution contained Tris buffer, diaminobenzidine, and H_2_O_2_.

### Affinity blotting

Affinity blot was performed using *P. tobbi* SGH separated and electrotransferred as described for Immunoblot. After transfer, free binding sites on NC membrane were blocked by 5% BSA in 20 mM Tris-Tw overnight at 4°C. The strip was then incubated for 1 h at room temperature with biotinylated lectin from *Canavalia ensiformis* (Concanavalin A, Sigma) diluted 0.2 µg/ml in Tris-Tw. To control the reaction specificity, another strip was incubated with lectin preincubated for 30 min with the ligand, 0.5 M methyl-α-D-mannopyranoside. Avidin-peroxidase (Sigma) was added at a final concentration of 2.5 µg/ml and incubated for 1 h at room temperature. Substrate solution contained Tris buffer, diaminobenzidine, and H_2_O_2_.

### Ethics statement

All animals used in this study were maintained and handled strictly in accordance with institutional guidelines and legislation for the care and use of animals for research purpose Czech Act No. 246/1992 coll. on Protection Animals against Cruelty in present statues at large that complies with all relevant European Union and international guidelines for experimental animals. The experiments were approved by the Committee on the Ethics of Animal Experiments of the Charles University in Prague (Permit Number: 24773/2008-10001) and were performed under the Certificate of Competency (Registration Number: CZU 934/05) in accordance with the Examination Order approved by Central Commission for Animal Welfare of the Czech Republic. All efforts were made to minimize suffering of experimental animals within the study.

## Results and Discussion

### Salivary gland transcripts analysis

*Phlebotomus tobbi* and *P. sergenti* cDNA libraries were constructed from SGs of female sand flies dissected one day after emergence. From each cDNA library, 1,152 randomly selected clones were sequenced. Obtained ESTs were deposited in the NCBI dbEST database under accession numbers GW814275–GW815416 (1,142 sequences) for *P. tobbi* and GW813185–GW814274 (1,090 sequences) for *P. sergenti*. High-quality sequences were grouped together based on sequence homology, and resulting assembled sequences were analyzed using the dCAS cDNA annotation software [38] and verified by manual annotation. In the *P. tobbi* cDNA library, 997 high-quality sequences were grouped into 68 contigs and 125 singletons (one sequence in cluster); in *P. sergenti*, 853 high-quality sequences were grouped into 56 contigs and 196 singletons.

**Figure 1:**
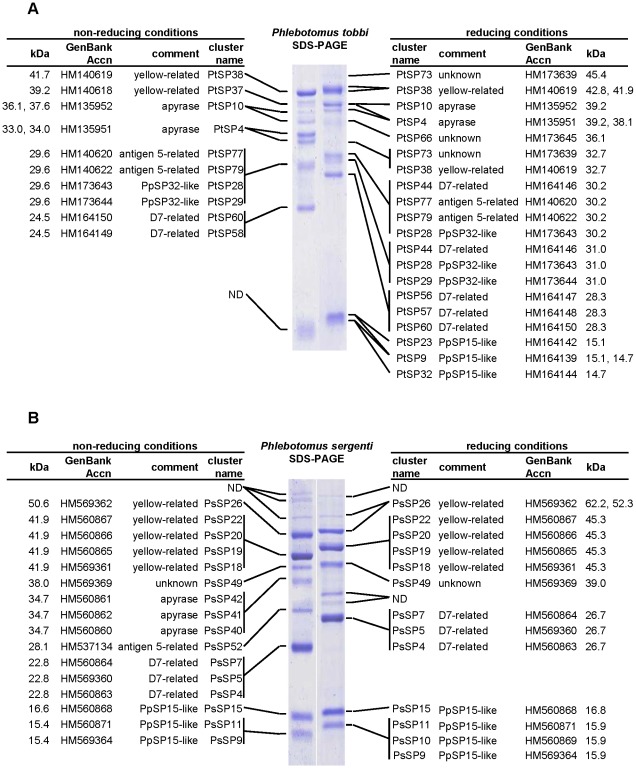
Proteome analysis of sand fly salivary proteins. (**A**) *Phlebotomus tobbi* and (**B**) *P. sergenti* salivary gland homogenate were separated under reducing and non-reducing conditions. Resulting protein bands were cut from Coomassie-stained gel and analyzed by mass spectrometry. Obtained data were compared to relevant cDNA library. Identified proteins are listed with their GenBank accession number, cluster name, and molecular weight of the protein band (kDa). ND means not determined due to insignificant results.

Similar to other sand flies studied so far, the most abundant transcripts in both libraries were those coding for putative secretory proteins. BLAST comparison of translated nucleotide sequences with the non-redundant (NR) protein database showed high similarity with other sand fly secreted salivary proteins. In *P. tobbi*, 81 clusters containing 863 sequences (average 10.7 sequences per cluster) matched to sand fly salivary proteins. Of them, we found 62 clusters (796 sequences) with predicted signal peptide sequence. In *P. sergenti*, 50 clusters containing 553 sequences (average 11.1 sequences per cluster) matched to sand fly salivary proteins. Of them, 32 clusters (482 sequences) with predicted signal protein sequence were found. Tables 1 and 2 list representative secreted salivary proteins from *P. tobbi* and *P. sergenti*, respectively, deposited into NCBI GenBank database. The tables show GenBank accession numbers, putative mature protein features, best match to NR protein database, and presence in the proteome analysis as confirmed by MS (Figure 1). Additionally, Figure 1A and 1B show detailed analysis of MS results for *P. tobbi* and *P. sergenti*, respectively, including cluster name, Gen Bank accession number, and molecular weight of mature proteins under reducing and non-reducing conditions.

**Table 1:** Salivary protein transcripts of *Phlebotomus tobbi*.

				Putative mature protein			Best match to NR protein database		
Cluster name	GenBank Accn	Comment	In proteome	pI	Mw	AA	GenBank Accn	Species	E Value
PtSP49	HM173648	41.9-kDa		8.3	45.5	410	ABA43063	*P. perniciosus*	0
PtSP38	HM140619	yellow-related	Yes	8.5	42.6	375	ABA43050	*P. perniciosus*	0
PtSP37	HM140618	yellow-related	Yes	6.0	41.5	370	ABA43049	*P. perniciosus*	0
PtSP73	HM173639	ParSP25-like	Yes	4.4	38.8	336	ABA43056	*P. perniciosus*	4E-87
PtSP10	HM135952	apyrase	Yes	9.1	35.7	311	ABB00906	*P. perniciosus*	1E-180
PtSP4	HM135951	apyrase	Yes	9.1	35.2	311	ABB00907	*P. perniciosus*	1E-174
PtSP66	HM173645	33-kDa	Yes	9.0	33.9	288	ABA43054	*P. perniciosus*	4E-155
PtSP77	HM140620	antigen 5-related	Yes	9.1	31.2	272	ABA43055	*P. perniciosus*	3E-151
PtSP78	HM140621	antigen 5-related		9.2	30.2	263	ABA43055	*P. perniciosus*	1E-149
PtSP79	HM140622	antigen 5-related	Yes	9.2	28.8	252	ABA43055	*P. perniciosus*	2E-151
PtSP76	HM173641	ParSP25-like		4.5	28.0	244	ABA43056	*P. perniciosus*	7E-95
PtSP75	HM173640	ParSP25-like		4.6	27.8	243	ABA43056	*P. perniciosus*	3E-77
PtSP56	HM164147	D7-related	Yes	8.1	27.1	233	ABA43051	*P. perniciosus*	1E-122
PtSP60	HM164150	D7-related	Yes	8.3	27.0	234	ABA43052	*P. perniciosus*	8E-119
PtSP54	HM164151	D7-related		8.3	27.0	233	ABA43051	*P. perniciosus*	3E-125
PtSP58	HM164149	D7-related	Yes	9.4	26.8	230	ABA43052	*P. perniciosus*	4E-117
PtSP44	HM164146	D7-related	Yes	8.9	26.7	233	ABA43058	*P. perniciosus*	5E-124
PtSP57	HM164148	D7-related	Yes	8.7	25.9	225	ABA43052	*P. perniciosus*	8E-116
PtSP42	HM164145	D7-related		9.5	25.3	216	ABA43058	*P. perniciosus*	1E-119
PtSP28	HM173643	PpSP32-like	Yes	10.0	24.5	227	ABA43053	*P. perniciosus*	1E-100
PtSP29	HM173644	PpSP32-like	Yes	10.1	24.5	227	ABA43053	*P. perniciosus*	4E-99
PtSP27	HM173642	PpSP32-like		10.1	24.3	225	ABA43053	*P. perniciosus*	7E-92
PtSP9	HM164139	PpSP15-like	Yes	8.6	14.9	122	ABA43048	*P. perniciosus*	2E-65
PtSP17	HM164140	PpSP15-like		8.0	14.7	123	AAX55748	*P. ariasi*	8E-40
PtSP32	HM164144	PpSP15-like	Yes	8.7	14.6	121	ABA43057	*P. perniciosus*	1E-69
PtSP31	HM164143	PpSP15-like		8.7	14.4	119	ABA43057	*P. perniciosus*	4E-51
PtSP18	HM164141	PpSP15-like		8.6	13.8	118	ABA43059	*P. perniciosus*	5E-54
PtSP23	HM164142	PpSP15-like	Yes	9.1	13.2	112	ABA43059	*P. perniciosus*	3E-63
PtSP8	HM173646	unknown		10.2	5.0	43	ABB00905	*P. perniciosus*	1.5
PtSP71	HM173638	unknown		10.6	4.5	42	ABA43060	*P. perniciosus*	5E-12
PtSP81	HM173647	unknown		9.5	3.7	34	ABB00905	*P. perniciosus*	5E-10
PtSP125	JN192442	hyaluronidase					ACS93505	*P. arabicus*	2E-61

Putatively secreted salivary proteins from *Phlebotomus tobbi* with cluster name, GenBank accession number (GenBank Accn), presence in the proteome analysis as confirmed by mass spectrometry (Figure 1A), putative mature protein features (pI, predicted isoelectric point; Mw, predicted molecular weight; AA, number of amino acid residues), and best match to non-redundant protein database.

**Table 2:** Salivary protein transcripts of *Phlebotomus sergenti*.

				Putative mature protein			Best match to NR protein database		
Cluster name	GenBank Accn	Comment	In proteome	pI	Mw	AA	GenBank Accn	Species	E Value
PsSP82	HM569371	41.9-kDa		4.74	56.6	508	ABI20189	*P. duboscqi*	1E-78
PsSP26	HM569362	yellow-related	Yes	8.06	43.9	382	ABI15938	*P. duboscqi*	0
PsSP19	HM560865	yellow-related	Yes	8.86	42.5	377	AAL11051	*P. papatasi*	1E-176
PsSP20	HM560866	yellow-related	Yes	9.80	42.4	377	AAL11051	*P. papatasi*	1E-178
PsSP22	HM560867	yellow-related	Yes	5.70	42.3	377	ABI20172	*P. duboscqi*	1E-164
PsSP18	HM569361	yellow-related	Yes	9.02	42.2	377	AAL11051	*P. papatasi*	1E-171
PsSP42	HM560861	apyrase	Yes	8.91	35.9	317	AAG17637	*P. papatasi*	1E-135
PsSP40	HM560860	apyrase	Yes	8.87	35.6	315	AAG17637	*P. papatasi*	1E-123
PsSP41	HM560862	apyrase	Yes	8.31	33.7	295	AAG17637	*P. papatasi*	1E-134
PsSP49	HM569369	33-kDa	Yes	9.00	32.9	279	ABI20155	*P. duboscqi*	1E-131
PsSP52	HM537134	antigen 5-related	Yes	8.75	29.0	254	ABA54266	*P. papatasi*	1E-121
PsSP4	HM560863	D7-related	Yes	8.93	26.8	233	AAL11048	*P. papatasi*	1E-101
PsSP5	HM569360	D7-related	Yes	8.93	26.8	233	AAL11048	*P. papatasi*	1E-102
PsSP7	HM560864	D7-related	Yes	8.41	26.7	233	AAL11048	*P. papatasi*	1E-102
PsSP44	HM569368	PpSP32-like		9.3	22.5	204	AAL11050	*P. papatasi*	1E-67
PsSP14	HM560870	PpSP15-like		8.76	17.1	142	AAL11047	*P. papatasi*	1E-40
PsSP15	HM560868	PpSP15-like	Yes	9.07	14.7	122	AAL11047	*P. papatasi*	3E-45
PsSP54	HM569365	PpSP15-like		8.61	14.6	121	AAL11046	*P. papatasi*	2E-52
PsSP55	HM569363	PpSP15-like		8.61	14.6	121	AAL11046	*P. papatasi*	3E-52
PsSP98	HM569366	unknown		4.73	14.3	127	ABA12153	*P. argentipes*	3E-16
PsSP9	HM569364	PpSP15-like	Yes	9.06	14.0	120	AAL11045	*P. papatasi*	4E-51
PsSP10	HM560869	PpSP15-like	Yes	8.92	14.0	120	AAL11045	*P. papatasi*	2E-52
PsSP11	HM560871	PpSP15-like	Yes	8.05	13.9	120	AAL11045	*P. papatasi*	7E-53
PsSP73	HM569367	unknown		4.51	12.2	118	AAX55657	*P. ariasi*	2E-20
PsSP28	HM569370	unknown		10.68	3.0	27	ABI20185	*P. duboscqi*	4E-6

Putatively secreted salivary proteins from *Phlebotomus sergenti* with cluster name, GenBank accession number (GenBank Accn), presence in the proteome analysis as confirmed by mass spectrometry (Figure 1B), putative mature protein features (pI, predicted isoelectric point; Mw, predicted molecular weight; AA, number of amino acid residues), and best match to non-redundant protein database.

The putative secreted salivary proteins of *P. tobbi* and *P. sergenti* could be divided into ten main protein families (Figure S1): antigen 5-related protein, apyrase, hyaluronidase, D7-related and PpSP15-like protein (odorant-binding proteins superfamily), ParSP25-like protein, PpSP32-like protein, yellow-related protein, the 33-kDa salivary proteins, and the 41.9-kDa superfamily. The following paragraphs describe these families in detail, focusing on protein family characteristics, possible function, biochemical, immunomodulatory, and antigenic properties, and phylogenetic analysis in context with related proteins from other sand flies.

### Antigen-5 related protein

Antigen 5-related proteins (Ag5r) are present in saliva of all sand fly species studied so far [55], [56], including *P. tobbi* (PtSP77/HM140620, PtSP78/HM140621, PtSP79/HM140622) and *P. sergenti* (PsSP52/HM537134). Sand fly Ag5r proteins are members of CAP superfamily consisting of mammalian Cysteine-rich secretory proteins (CRISPs), Antigen 5 (Ag5) originally described from wasp venom, and plant Pathogenesis-related 1 proteins (PR-1) [55]. Proteins with CAP domain occur across all living organisms, including prokaryotes [57], and are mostly extracellular/secreted.

**Figure 2:**
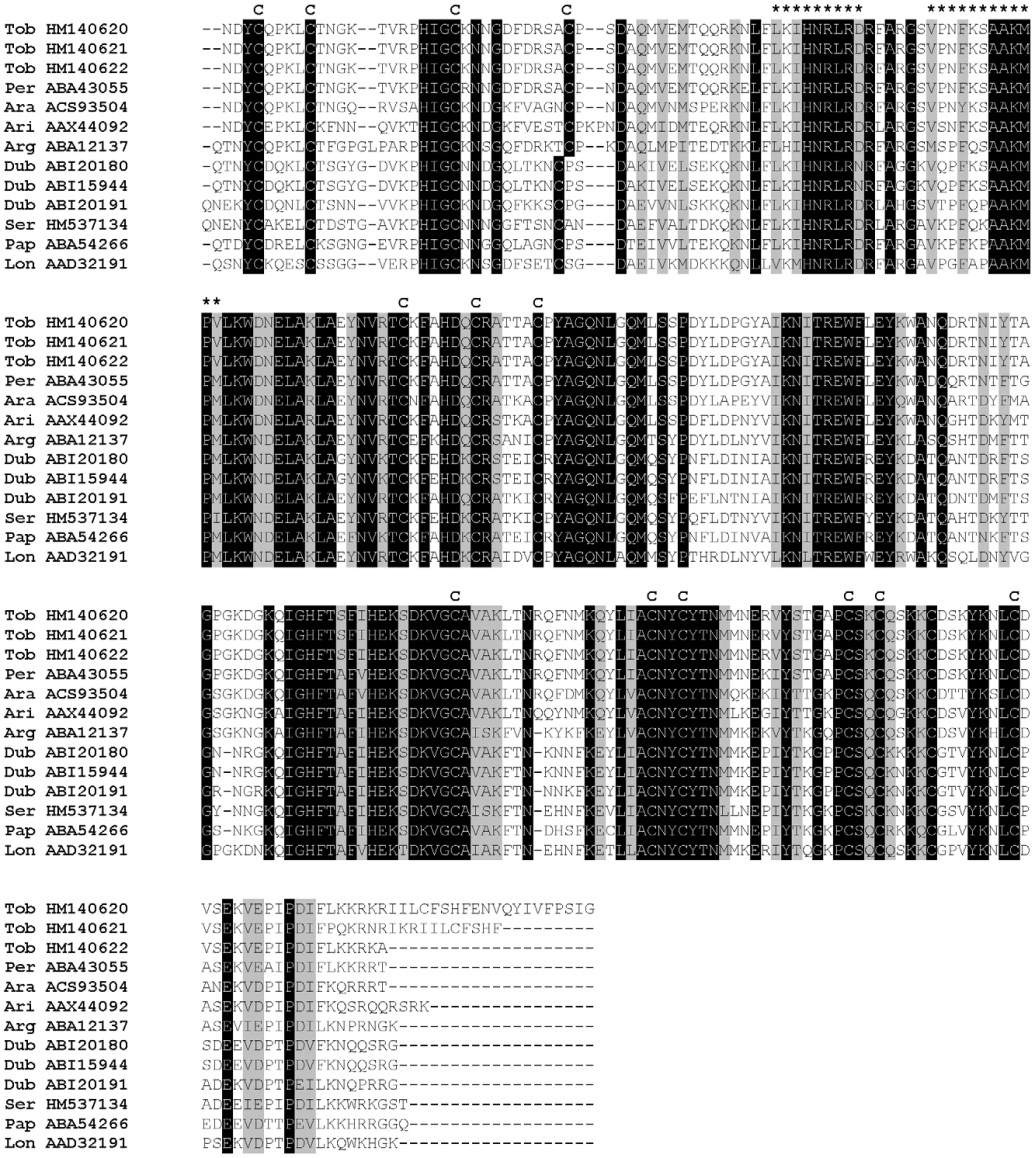
Multiple sequence alignment of the antigen 5-related family of salivary proteins. Multiple sequence alignment of sand fly antigen 5-related proteins from *Phlebotomus arabicus* (Ara), *P. argentipes* (Arg), *P. ariasi* (Ari), *P. duboscqi* (Dub), *P. papatasi* (Pap), *P. perniciosus* (Per), *P. sergenti* (Ser), *P. tobbi* (Tob), and *Lutzomyia longipalpis* (Lon). Sequences without signal peptide were aligned using ClustalX and manually refined using BioEdit sequence-editing software. Accession numbers are indicated in the sequence name. Identical amino acid residues are highlighted black and similar residues grey. Conserved cysteine residues are indicated above the alignment by letter C and T cell epitopes predicted for *P. duboscqi* by Kato et al. [26] are indicated by asterisk (*).

All sand fly Ag5r proteins have similar predicted molecular mass (ranging from 28.8 to 31.2 kDa) and are alkaline (Table S1). In *P. tobbi* and *P. sergenti*, the predicted molecular mass corresponded well with the one measured in proteomic analysis (Figure 1, Tables 1 and 2) suggesting single-domain protein and negligible post-translational modifications. We identified 14 highly conserved cysteine residues proportionally distributed through the whole sequence length (Figure 2), possibly involved in disulfide bonding.

Although the members of this family were described in sialotranscriptomes of all bloodsucking arthropods characterized [55], [56], their role is mostly unknown with a few exceptions. In *Stomoxys calcitrans*, Ag5r protein possesses immunoglobulin Fc binding activity [58]. In *Tabanus yao*, members of the Ag5r protein family can probably serve as an inhibitors of angiogenesis (RTS disintegrin motif) [59] or a potent platelet inhibitor (RGD motif) [60]. The Ag5r proteins are not specific for salivary glands thus they may possess other functions not associated with feeding [23], [56].

Several studies showed antigenic properties associated with Ag5r proteins. Plasmid coding for Ag5r protein from *P. ariasi* (ParSP05/AAX44092) induced a cell-mediated immune response in Swiss Webster mice [27], showing that sand fly Ag5r proteins might modulate cell-mediated host immune response. This presumption is also supported by several T cell epitopes predicted for *P. duboscqi* Ag5r proteins [26] that include regions highly conserved among sand flies (Figure 2). Antibody response to sand fly Ag5r proteins was demonstrated in *P. perniciosus*; Ag5r protein (PpeSP07/ABA43055) reacted with IgG antibodies from sera of *P. perniciosus* bitten dogs [21]. In other bloodsucking diptera, Ag5r proteins are mostly associated with IgE antibody response. Ag5r protein of *Simulium vittatum* seems to be the major allergen for insect bite hypersensitivity sharing common IgE-binding epitopes with Ag5r protein from *Culicoides nubeculosus* [61], [62]. Specific anti-Ag5r IgE antibodies were also observed in Ugandan individuals bitten by *Glossina morsitans* [63].

**Figure 3:**
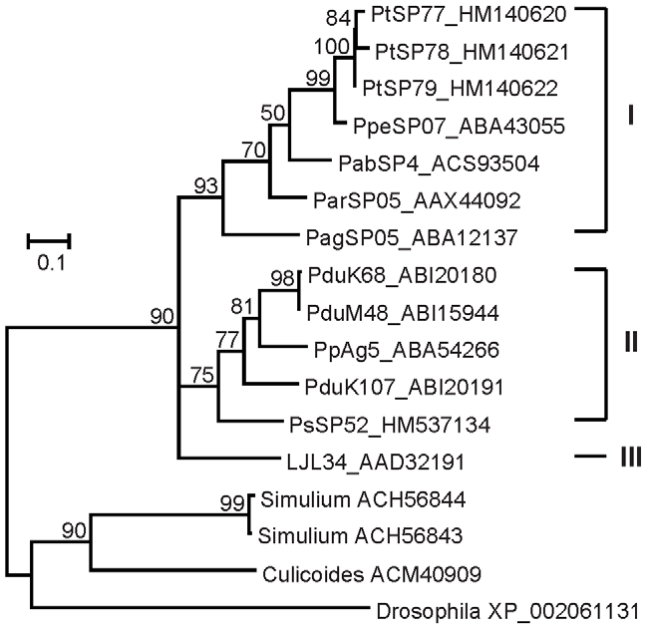
Phylogenetic analysis of the antigen 5-related family of sand fly salivary proteins. Phylogenetic analysis of antigen 5-related proteins from *Phlebotomus arabicus* (Pab), *P. argentipes* (Pag), *P. ariasi* (Par), *P. duboscqi* (Pdu), *P. papatasi* (Pp), *P. perniciosus* (Ppe), *P. sergenti* (Ps), *P. tobbi* (Pt), *Lutzomyia longipalpis* (LJL), and antigen 5 sequences from *Simulium vittatum*, *Culicoides nubeculosus*, and *Drosophila willistoni*. Phylogenetic analysis was conducted on amino acid sequences without signal peptide using Tree Puzzle (version 5.2) by maximum likelihood (WAG model), quartet puzzling, and automatically estimated internal branch node support (10,000 replications). Sequence names, accession numbers, and branch node values are indicated.

Phylogenetic analysis of Ag5r proteins from sand flies and other insects showed a strongly supported distinct clade of sand fly Ag5r proteins (Figure 3) similar to a previous analysis by [28]. The relationship within the sand fly clade reflected phylogenetic relationship within phlebotomine sand flies [33], showing three distinct branches: clade I with species belonging to subgenera *Euphlebotomus*, *Larroussius*, and *Adlerius*; clade II with *Phlebotomus* and *Paraphlebotomus* species (*P. papatasi*, *P. duboscqi*, and *P. sergenti*); and *Lutzomyia* in clade III (Figure 3).

### Apyrase

Apyrase (EC 3.6.1.5) appears to be a universal enzyme used to prevent blood coagulation by diverse hematophagous animals such as bloodsucking leeches, ticks, triatomine bugs, fleas, and mosquitoes. This enzyme hydrolyses both ATP and ADP to AMP, thus destroying an important physiologic stimulus of platelet aggregation released from damaged tissues and blood cells. Apyrases of bloodsucking insects are divided into three families: CD-39 (the actin/heat shock 70/sugar kinase superfamily); 5′-nucleotidase; and *Cimex*-type [55], [56].

Sand flies are not an exception; transcripts coding for apyrases have been found in the saliva of all tested species [6], [23]–[28], including *P. tobbi* (PtSP4/HM135951, PtSP10/HM135952) and *P. sergenti* (PsSP40/HM560860, PsSP41/HM560862, PsSP42/HM560861) (Tables 1 and 2). The predicted molecular mass of the translated molecules is uniform for all sand fly species, varying between 35 and 36 kDa (Table S1). All sand fly apyrases deposited in GenBank have also been found in the proteomic analysis (Table S1). In *P. tobbi* and *P. sergenti*, the predicted molecular mass corresponds well with the molecular weight measured under non-reducing conditions (33.0–37.6 kDa) (Figure 1; Tables 1 and 2).

Sand fly apyrases belong to the *Cimex*-type apyrase family. They hydrolyze ADP at a faster rate than ATP [64] and, similar to *Cimex lectularius*, the activity strictly depends on Ca^2+^ but not Mg^2+^ ions [6], [23], [64]–[66]. Apyrase activity has been demonstrated in the saliva of *L. longipalpis* [23], [64], *P. argentipes* [65], *P. colabaensis* [65], *P. duboscqi* [66] *P. papatasi* [65], [67], *P. perniciosus* [65], and as well as in recombinant apyrases of *P. papatasi* (PpApy/AF261768) [67] and *P. duboscqi* (PduApy2/DQ834331) [66]. Bacterially expressed *P. duboscqi* apyrase inhibited ADP- as well as collagen-induced platelet aggregation [66], indicating that post-translational modifications such as glycosylation are not necessary for apyrase activity.

**Figure 4:**
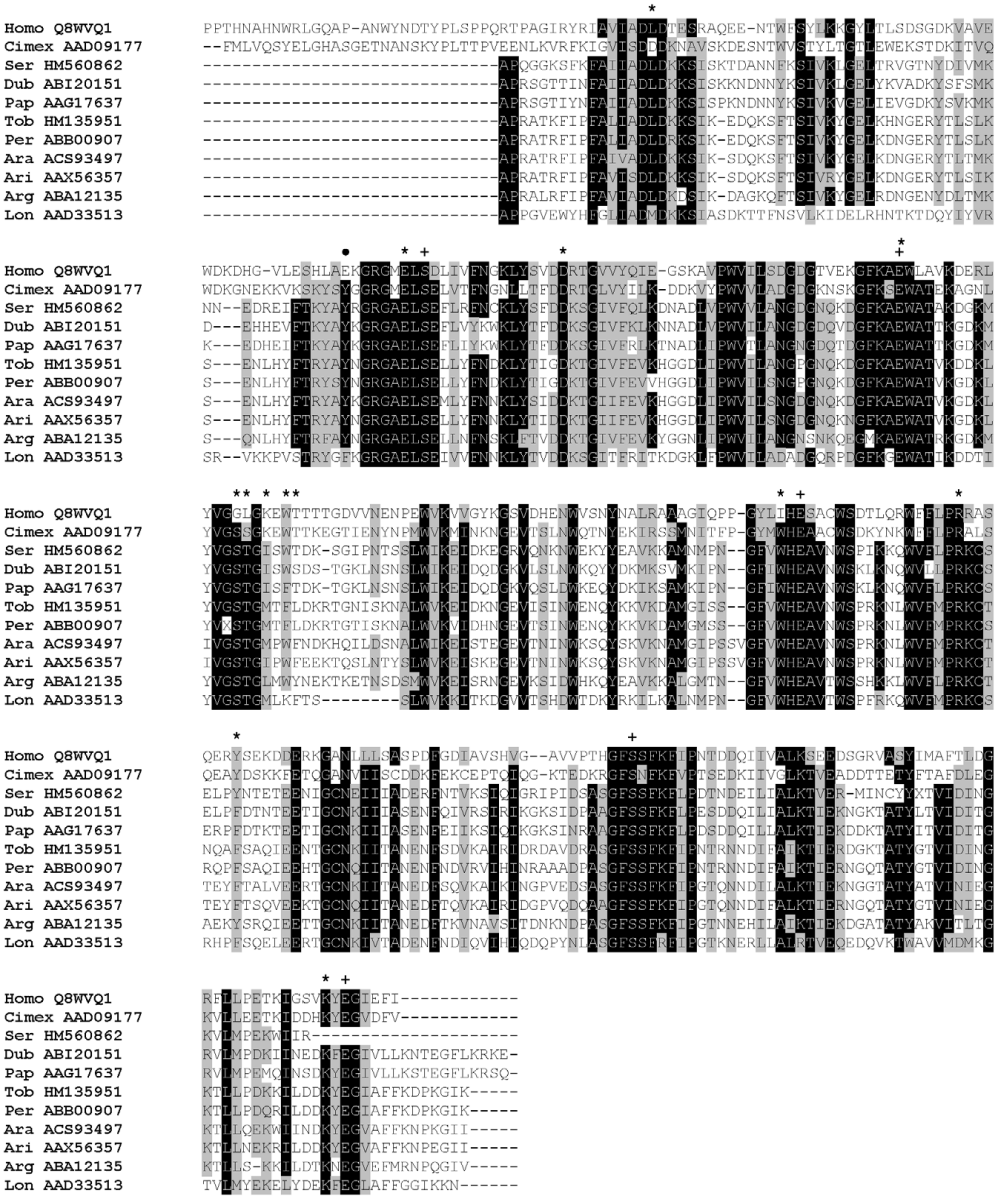
Multiple sequence alignment of the apyrase family of salivary proteins. Multiple sequence alignment of sand fly apyrases from *Phlebotomus arabicus* (Ara), *P. argentipes* (Arg), *P. ariasi* (Ari), *P. duboscqi* (Dub), *P. papatasi* (Pap), *P. perniciosus* (Per), *P. sergenti* (Ser), *P. tobbi* (Tob), *Lutzomyia longipalpis* (Lon), and related sequences from *Cimex lectularius* and *Homo sapiens*. Sequences without signal peptide were aligned using ClustalX and manually refined using BioEdit sequence-editing software. Accession numbers are indicated in the sequence name. Identical amino acid residues are highlighted black and similar residues grey. Nucleotide binding sites (*) and Ca^2+^ binding sites (+), as predicted for human apyrase by Dai et al. [70], are indicated. The position of E92Y point mutation of human apyrase described by Yang and Kirley [69] is indicated by (•).

Orthologs of the Cimex apyrase family have also been identified in vertebrates and termed calcium-activated nucleotidases (CANs) [68]. In contrast to sand flies, human soluble CAN-1 (SCAN-1) preferentially hydrolyses UDP and GDP; however, the engineered SCAN-1 mutant Glu92Tyr shows five times and seven times higher hydrolysis activity for ADP and ATP, respectively [69]. This mutated tyrosine is conserved among species of the genus *Phlebotomus* (Figure 4), supporting its key role in substrate specificity for phlebotomine apyrases [69]. In human SCAN-1, other amino acid residues essential for binding nucleotide and Ca^2+^ were identified [70], some of them being absolutely conserved among the analyzed apyrase proteins (Asp44, Ser100, Asp114, Glu216, Arg232, Ser277), while others were uniformly mutated within sand fly apyrases (Asp101Glu, Gly160Ser, Ile214Trp) (Figure 4).

Besides hydrolyzing activity, sand fly apyrases also possess antigenic properties. Antibodies from dogs experimentally or naturally exposed to *P. perniciosus* strongly recognized PpeSP01 (ABB00906) and PpeSP01B (ABB00907) apyrases [21]. In humans naturally exposed to sand flies, anti-sand fly saliva IgG antibodies recognized a protein band corresponding, in molecular weight, to apyrase [11], [12]. Moreover, antibodies elicited by *P. duboscqi* saliva also recognized bacterially expressed *P. duboscqi* apyrase [66], indicating that not all antibodies are specific for possible glycan modifications of sand fly apyrases.

**Figure 5:**
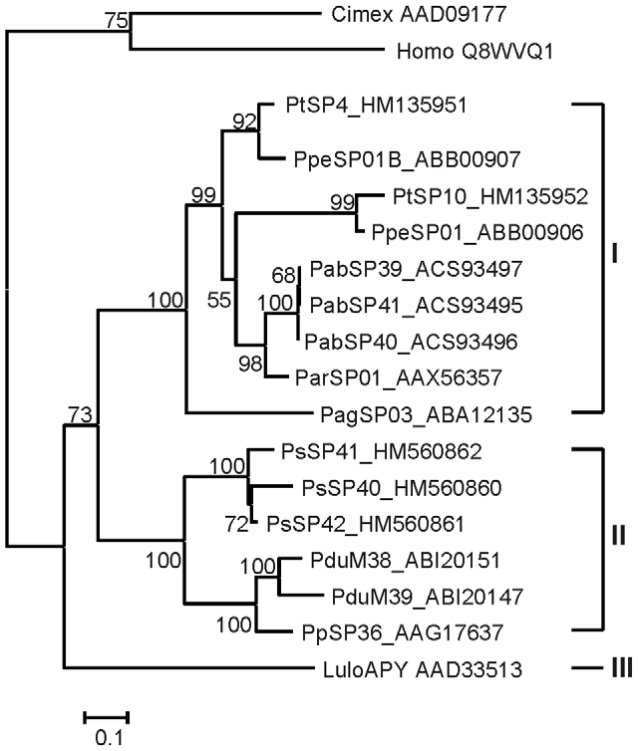
Phylogenetic analysis of the apyrase family of sand fly salivary proteins. Phylogenetic analysis of apyrases from *Phlebotomus arabicus* (Pab), *P. argentipes* (Pag), *P. ariasi* (Par), *P. duboscqi* (Pdu), *P. papatasi* (Pp), *P. perniciosus* (Ppe), *P. sergenti* (Ps), *P. tobbi* (Pt), *Lutzomyia longipalpis* (Lulo), and related apyrase sequences from *Cimex lectularius* and *Homo sapiens*. Phylogenetic analysis was conducted on amino acid sequences without signal peptide using Tree Puzzle (version 5.2) by maximum likelihood (WAG model), quartet puzzling, and automatically estimated internal branch node support (10,000 replications). Sequence names, accession numbers, and branch node values are indicated.

Phylogenetic analysis of sand fly apyrases reflects the same taxonomic relationship as Ag5r proteins. Figure 5 shows three distinct clades separating species in clade I (*P. arabicus*, *P. argentipes*, *P. ariasi*, *P. perniciosus*, *P. tobbi*) from *Phlebotomus* and *Paraphlebotomus* subgenera in clade II (*P. papatasi*, *P. duboscqi*, and *P. sergenti*), and genus *Lutzomyia* in clade III. This analysis showed a very close relationship within the *Larroussius* species, *P. tobbi* and *P. perniciosus* (Figure 5).

### Hyaluronidase

Hyaluronidase is an enzyme that catalyzes the hydrolysis of hyaluronic acid, a major component of the extracellular matrix in vertebrates. It is an ubiquitous enzyme found in mammals, bacteria and in the venom of bees, wasps, spiders, and snakes [71]. In bloodsucking Diptera, hyaluronidase activity has been found primarily in the saliva of telmophagic insects: horse flies, black flies, biting midges, and sand flies [72]. Thus, hyaluronidase is believed to decreases host skin tissue viscosity, assisting other salivary components to diffuse and create a pool of blood [60], [72], [73].

**Figure 6:**
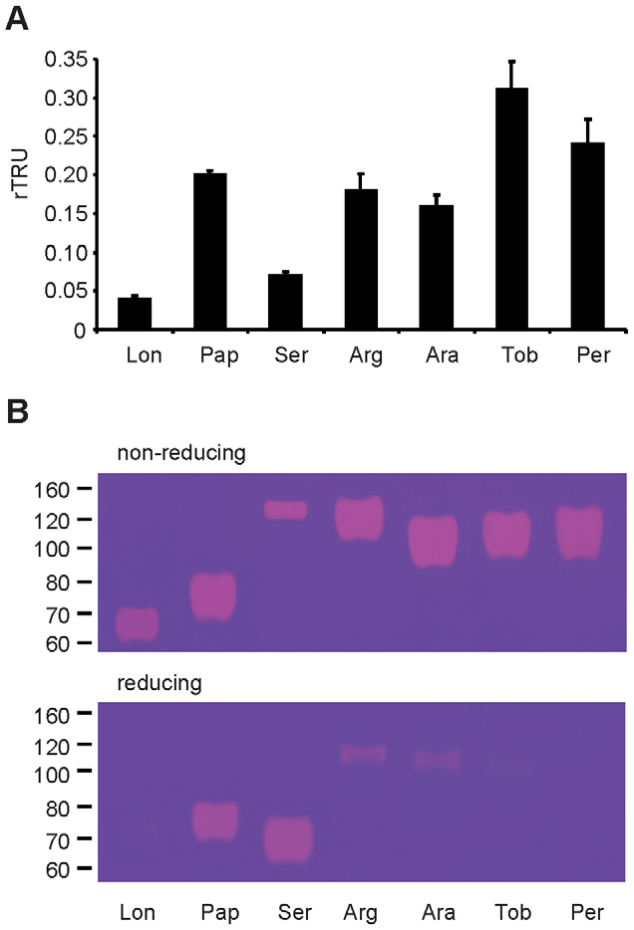
Comparison of hyaluronidase activity in seven sand fly species. (**A**) Hyaluronidase activity was compared in the same species using salivary gland homogenate equivalent to 0.5 gland using the microtitration plate method. The results are expressed in relative Turbidity Reducing Units ± standard error, using bovine testicular hyaluronidase as a standard: *L. longipalpis* = 0.04±0.001 rTRU, *P. papatasi* = 0.20±0.01 rTRU, *P. sergenti* (Israel) = 0.07±0.001 rTRU, *P. argentipes* = 0.18±0.02 rTRU, *P. arabicus* = 0.16±0.01 rTRU, *P. tobbi* = 0.31±0.04 rTRU, *P. perniciosus* = 0.24±0.03 rTRU. Three independent experiments were done. (**B**) SDS-PAGE zymography assay under reducing and non-reducing conditions on 8% polyacrylamide gel with incorporated hyaluronan for detection of hyaluronidase activity in salivary gland homogenate of seven sand fly species: *Lutzomyia longipalpis* (Lon), *Phlebotomus papatasi* (Pap), *P. sergenti* (Ser), *P. argentipes* (Arg), *P. arabicus* (Ara), *P. tobbi* (Tob), and *P. perniciosus* (Per).

Sand fly hyaluronidase belongs to the same family as mammalian and Hymenopteran hyaluronidases (endo-β-N-acetyl-hexosaminidases, E.C. 3.2.1.35) and is different from that of bloodsucking leeches and nematodes (endo-β-glucuronidases, E.C. 3.2.1.36) [71], [74]. Hyaluronidase activity has been detected in all eight sand fly species studied to date ([23], [28], [53], [73], Figure 6). Our zymographic analyses of *P. tobbi* (Figure 6) and *P. sergenti* originating from Israel (Figure 6) and Turkey [53] showed the potent activity of sand fly hyaluronidase. Based on the microplate method, *P. tobbi* hyaluronidase activity is one of the highest measured (Figure 6A). In contrast, hyaluronidase of *P. sergenti* had the lowest activity among the species of the genus *Phlebotomus* (Figure 6A). Under non-reducing conditions, *P. tobbi* and *P. sergenti* hyaluronidase revealed diffuse bands with the molecular weight of around 110 and 135 kDa, respectively (Figure 6B). Hyaluronidase of *P. sergenti* is probably a homodimer, because under reducing conditions, the activity was observed at about half of the molecular weight, both in the Israeli (Figure 6B) and Turkish strains [53], while hyaluronidase of *P. tobbi* was monomeric with similar molecular weight under non-reducing and reducing conditions and the activity reduced to minimum when denaturated and treated with β-mercaptoethanol (Figure 6B). Similar features were observed for the hyaluronidase of *P. perniciosus*, the other *Larroussius* species ([53], Figure 6), which suggests common biochemical characteristics of this enzyme between closely related species. In general, the remarkably high activity of salivary hyaluronidase may aid the spread of other salivary components as well as transmitted pathogens. Indeed, hyaluronidase coinjected with *Le. major* promotes infection in BALB/c mice [72]; however, no association was found between hyaluronidase activity and the sand fly capacity to vector either cutaneous or visceral leishmaniasis (Figure 6A).

**Figure 7:**
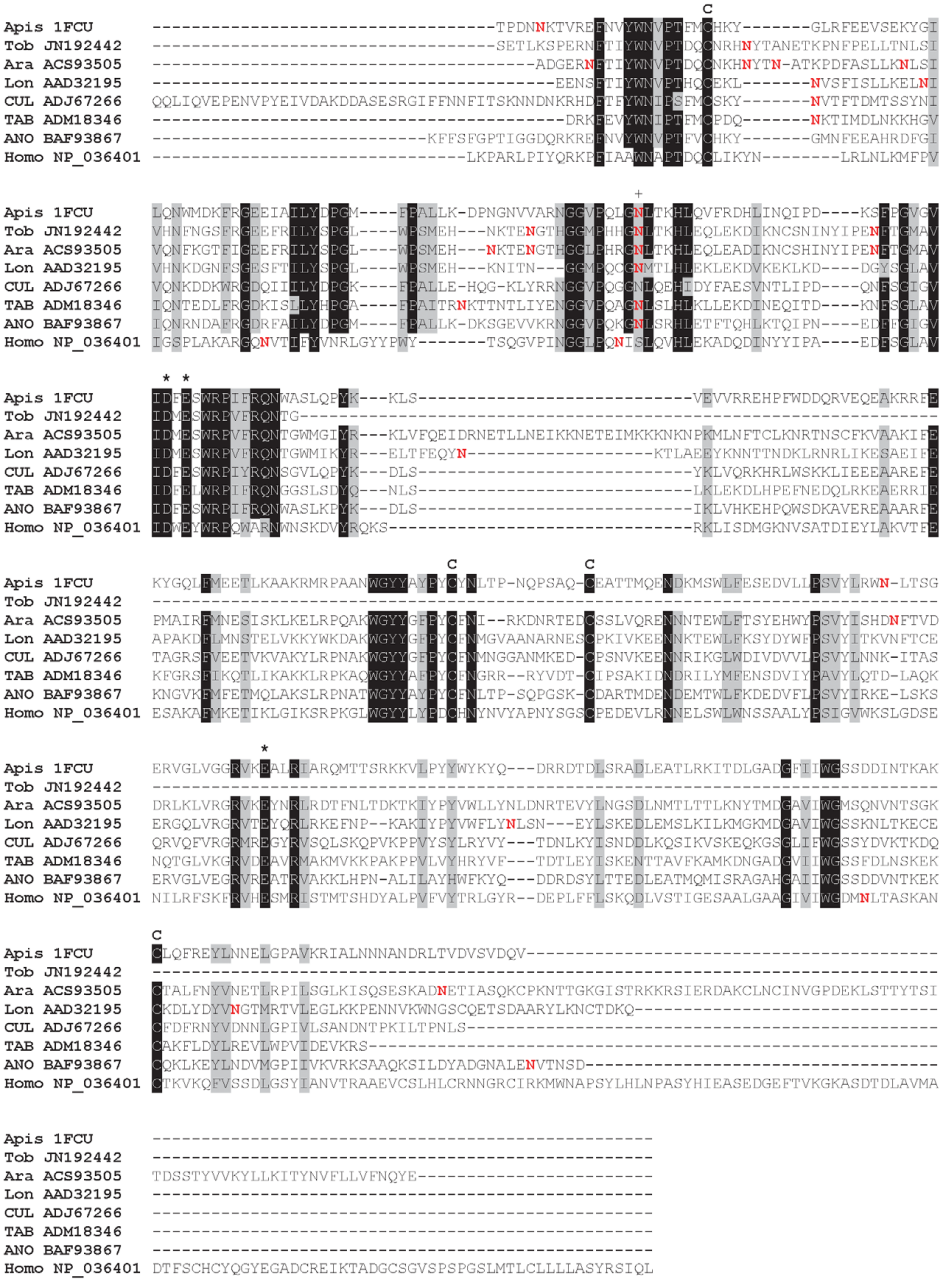
Multiple sequence alignment of the hyaluronidase family of salivary proteins. Multiple sequence alignment of hyaluronidases from *Phlebotomus arabicus* (Ara), *P. tobbi* (Tob), *Lutzomyia longipalpis* (Lon), and related sequences from *Apis mellifera*, *Culicoides nubeculosus* (CUL), *Tabanus yao* (TAB), *Anoplius samariensis* (ANO), and *Homo sapiens*. Sequences without signal peptide were aligned using ClustalX and manually refined using BioEdit sequence-editing software. Accession numbers are indicated in the sequence name. Identical amino acid residues are highlighted black and similar residues grey. Active site residues (*) and cysteine residues forming disulfide bridges (C) as predicted for *Apis* hyaluronidase by Markovic-Housley et al. [75] are indicated. Red residues (N) denote predicted N-glycosylation sites, including one (+) highly conserved among aligned sequences.

Although sand fly hyaluronidase is a very potent enzyme, it is scarcely found in transcriptomic and proteomic approaches probably due to the low abundance of transcripts combined with the large size of the protein. Hyaluronidase transcripts have been reported in only two of seven salivary cDNA libraries, namely in *L. longipalpis* and *P. arabicus* [23], [24], [28]. In *P. sergenti*, no transcript was found, and in *P. tobbi*, only one 3′-truncated transcript was identified (PtSP125/JN192442). Amino acid residues that constitute the catalytic site (Asp111, Glu113, and Glu247) and form disulfide bridges (Cys22–Cys313 and Cys189–Cys201) in bee hyaluronidase [75] are conserved among the sand fly hyaluronidase sequences (Figure 7). Based on the NetNGlyc prediction server [48], several putative glycosylation sites were predicted in sand fly hyaluronidases, including one highly conserved among aligned sequences (Figure 7).

Allergenic properties of sand fly hyaluronidase are not known, although it has been identified or suspected as the main allergen in the saliva of other bloodsucking Diptera, namely biting midges and horseflies [59], [76]. However, there is no record of typical IgE-mediated allergic reaction to sand fly saliva; only negligible amount of anti-saliva IgE was measured in hosts repeatedly bitten by sand flies [11], [19], [77].

### Odorant binding-related proteins

Two sand fly salivary protein families, D7-related proteins and PpSP15-like proteins, are related to the arthropod pheromone/odorant binding protein superfamily (OBP, protein domain PBP-GOBP, pfam01395) [56], [78].

**Figure 8:**
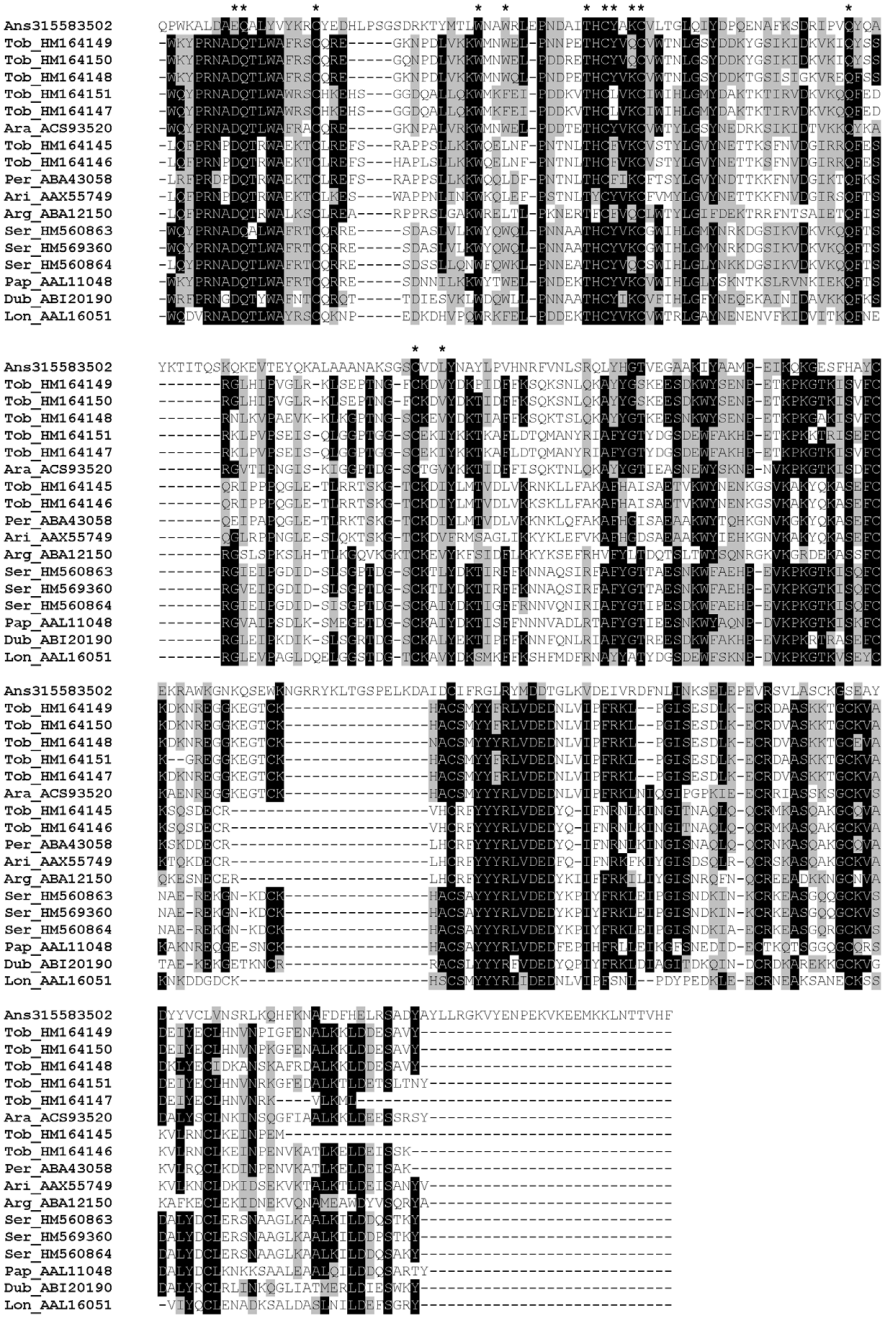
Multiple sequence alignment of the D7-related family of salivary proteins. Multiple sequence alignment of sand fly D7-related proteins from *Phlebotomus arabicus* (Ara), *P. argentipes* (Arg), *P. ariasi* (Ari), *P. duboscqi* (Dub), *P. papatasi* (Pap), *P. perniciosus* (Per), *P. sergenti* (Ser), *P. tobbi* (Tob), *Lutzomyia longipalpis* (Lon), and related sequence from *Anopheles stephensi* (Ans). Sequences without signal peptide were aligned using ClustalX and manually refined using BioEdit sequence-editing software. Accession numbers are indicated in the sequence name. Identical amino acid residues are highlighted black and similar residues grey. The cysteinyl leukotriene binding motif [56] is indicated by (*).

D7-related (D7r) proteins are named after the D7 protein, originally described in *Aedes aegypti* as a major salivary protein exclusively synthesized in bloodsucking females [79], [80]. Salivary proteins related to D7 have also been found in black flies and biting midges [56], [78] and all sand fly species studied to date. In the *P. tobbi* SG cDNA library we found seven clusters homologous to D7r sequences (HM164145–HM164151) and three clusters in the *P. sergenti* cDNA library (PsSP4/HM560863, PsSP5/HM569360, PsSP7/HM560864) (Tables 1 and 2). Within all sand flies, D7r proteins have similar predicted molecular mass (25.3–28.1 kDa) and wide range of pI (4.82–9.5) (Table S1). Based on the results from NetNGlyc prediction server [48], we found a mixture of putative glycosylated and non-glycosylated D7r sequences in most of the sand fly species studied with the exception of *L. longipalpis*, *P. sergenti*, and *P. papatasi*, where no N-glycosylation sites were found (Figure 8). All sand fly D7r predicted proteins contain nine highly conserved cysteine residues (Figure 8), implying there is a single non-disulphide-bond-forming cysteine.

The other family related to OBPs, PpSP15-like proteins, is closely related to larger D7-related proteins [25], [56] and are named after 15-kDa salivary protein of *P. papatasi* (PpSP15/AF335487) [6]. They have not been identified in any Diptera other than sand flies [25], [56]. It is the most abundant family among sand fly salivary proteins, and *P. tobbi* and *P. sergenti* are not exceptions; six and seven members of this family were found in each cDNA library, respectively (Tables 1 and Table 2). Several members were also detected by proteomic analysis, having similar molecular mass as predicted based on the amino acid sequences (Tables 1 and Table 2; Figure 1). Within the sand flies, PpSP15-like proteins have a similar predicted molecular mass (12.2–17.1 kDa) and surprisingly wide range of pI (6.33–9.44) (Table S1). In accordance with previous reports [25], [28], all sand fly PpSP15-like proteins show high degree of variability of around six conserved cystine residues (Figure S2).

In mosquitoes, some salivary D7 strongly bind biogenic amines and leukotrienes as well as components of the coagulation cascade, thus promptly antagonizing the host defense system [81]–[83]. D7r and PpSP15-like sand fly salivary proteins have not yet been characterized functionally; however, the motif [ED]-[EQ]-x(7)-C-x(12,17)-W-x(2)-W-x(7,9)-[TS]-x-C-[YF]-x-[KR]-C-x(8,22)-Q-x(22,32)-C-x(2)-[VLI], found in mosquito D7 salivary proteins that bind cysteinyl leukotrienes [83], is also found in the sand fly D7r proteins (Figure 8).

Sand fly PpSP15-like proteins and D7r proteins possess antigenic properties. PpSP15-like proteins were reported as promising anti-*Leishmania* vaccine candidates [6], [27], [84]. *Phlebotomus papatasi* SP15 protein is able to protect mice against *Le. major* challenge, and a DNA vaccine containing the PpSP15 cDNA provided the same protection [6]. ParSP03 (AAX56359), a PpSP15-like protein from *P. ariasi*, elicited similar delayed-type hypersensitivity and humoral immune responses upon DNA vaccination [27].

D7r could serve as a marker of exposure to sand fly bites. In humans, all tested serum samples from individuals naturally exposed to *P. papatasi* strongly bound to a *P. papatasi* protein band with a molecular mass corresponding to PpSP30 D7r protein (AAL11049) [12], [18]. As an ideal marker of exposure, this protein was recognized by both IgE and IgG antibodies, including all tested IgG subclasses [18]. D7r proteins seem to be applicable also for measurement of dog exposure, the main reservoir host for visceral leishmaniasis, since IgG antibodies from animals bitten by *P. perniciosus* [21] or *L. longipalpis* [16], [85] recognized D7r proteins of the respective species (PpeSP4/DQ150623, PpeSP04B/DQ150624, PpeSP10/DQ153104, LJL13/AF420274). Moreover, *L. longipalpis*-bitten dogs bind also to the LJL13 D7r recombinant form [16].

**Figure 9:**
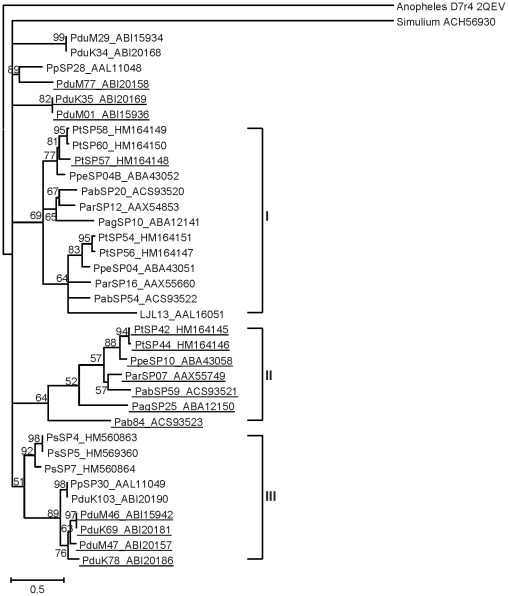
Phylogenetic analysis of the D7-related family of sand fly salivary proteins. Phylogenetic analysis of D7-related proteins from *Phlebotomus arabicus* (Pab), *P. argentipes* (Pag), *P. ariasi* (Par), *P. duboscqi* (Pdu), *P. papatasi* (Pp), *P. perniciosus* (Ppe), *P. sergenti* (Ps), *P. tobbi* (Pt), *Lutzomyia longipalpis* (LJL), and related sequences from *Anopheles gambiae* (D7r4) and *Simulium vittatum*. Phylogenetic analysis was conducted on amino acid sequences without signal peptide using Tree Puzzle (version 5.2) by maximum likelihood (WAG model), quartet puzzling, and automatically estimated internal branch node support (10,000 replications). Sequence names, accession numbers, and branch node values are indicated. Underlined sequences possess predicted N-glycosylation sites.

**Figure 10:**
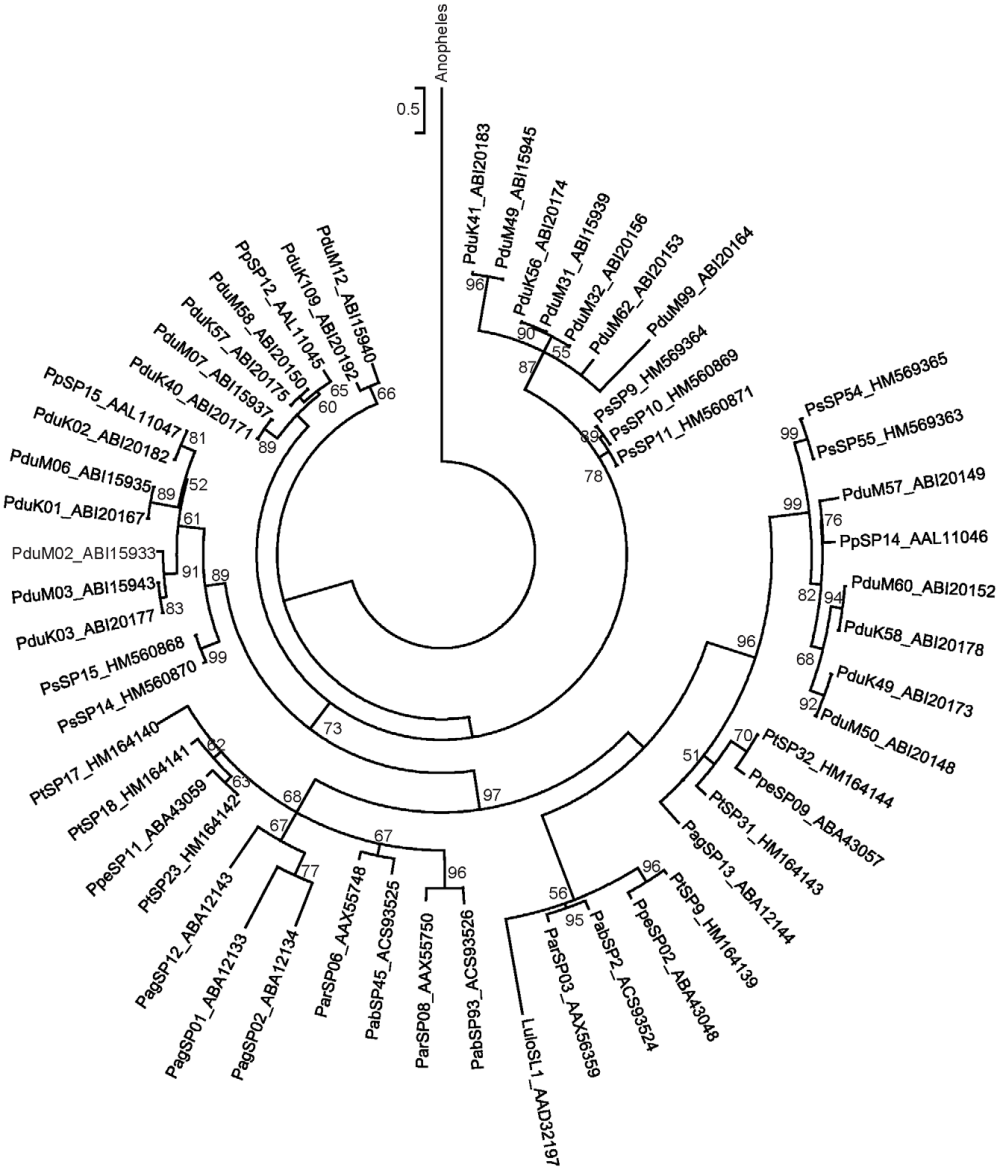
Phylogenetic analysis of the PpSP15-like family of sand fly salivary proteins. Phylogenetic analysis of the PpSP15-like proteins from *Phlebotomus arabicus* (Pab), *P. argentipes* (Pag), *P. ariasi* (Par), *P. duboscqi* (Pdu), *P. papatasi* (Pp), *P. perniciosus* (Ppe), *P. sergenti* (Ps), *P. tobbi* (Pt), *Lutzomyia longipalpis* (Lulo), and related sequence from *Anopheles gambiae* (XP_551869). Phylogenetic analysis was conducted on amino acid sequences without signal peptide using Tree Puzzle (version 5.2) by maximum likelihood (WAG model), quartet puzzling, and automatically estimated internal branch node support (10,000 replications). Sequence names, accession numbers, and branch node values are indicated.

Phylogenetic analysis of D7r proteins showed several major clades (Figure 9). *Phlebotomus sergenti* sequences clustered together forming a distinct subclade within clade III that contains *P. papatasi* and *P. duboscqi*. In contrast, *P. tobbi* D7r protein sequences are divided among clades I and II, which contain sequences from *P. arabicus*, *P. ariasi*, *P. argentipes*, *P. perniciosus*, and *L. longipalpis*. Interestingly, clade II only contained sequences with predicted N-glycosylation sites, which may suggest a unique functional characteristic of D7 molecules within this clade that have arisen after gene duplication. Similarly, phylogenetic analysis of PpSP15-like proteins (Figure 10) revealed several separated groups, consistently clustering *P. sergenti* sequences with *P. duboscqi* and *P. papatasi*, and *P. tobbi* sequences with those from *P. perniciosus* and other sand flies studied to date, including a single member from *L. longipalpis*. PpSP15 could be a multicopy gene, as more than two alleles were found in several *P. papatasi* individuals, some of them unique to the population origin [86].

### PpSP32-like proteins

**Figure 11:**
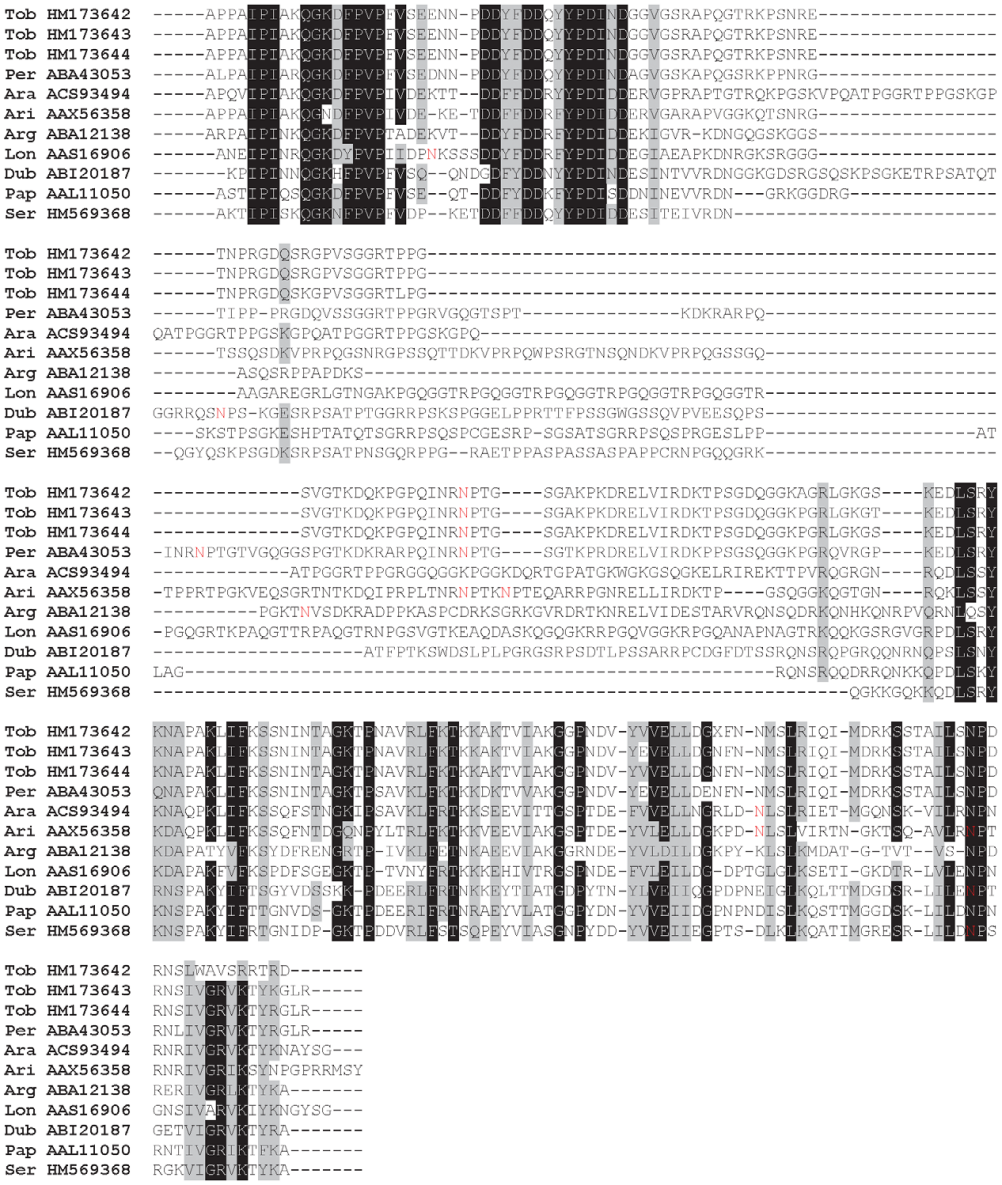
Multiple sequence alignment of the PpSP32-like family of salivary proteins. Multiple sequence alignment of sand fly PpSP32-like proteins from *Phlebotomus arabicus* (Ara), *P. argentipes* (Arg), *P. ariasi* (Ari), *P. duboscqi* (Dub), *P. papatasi* (Pap), *P. perniciosus* (Per), *P. sergenti* (Ser), *P. tobbi* (Tob), and *Lutzomyia longipalpis* (Lon). Sequences without signal peptide were aligned using ClustalX and manually refined using BioEdit sequence-editing software. Accession numbers are indicated in the sequence name. Identical amino acid residues are highlighted black and similar residues grey. Red residues (N) denote predicted N-glycosylation sites.

This family is named PpSP32 from the original identification in *P. papatasi* (AAL11050) [24] and due to the lack of homology to a conserved protein domain. PpSP32-like proteins have been described solely in sand flies and are found in all species studied so far; we identified homologous sequences also in *P. tobbi* (PtSP27/HM173642, PtSP28/HM173643, PtSP29/HM173644) and *P. sergenti* (PsSP44/HM569368). The predicted molecular mass of *P. tobbi* PpSP32-like proteins (24.5 kDa) is slightly lower than what was measured in proteomic analysis (Figure 1, Tables 1 and 2). All sequences have a wide range of predicted molecular mass (ranging from 22.5 to 34.9 kDa), no protein domain match, and are alkalic (pI ranging from 9.3 to 10.6) (Table S1). An interesting common feature of this protein family is that it possesses highly conserved N- and C- terminal regions with extremely variable internal sequence (Figure 11). Within the genus *Phlebotomus* there are predicted N-glycosylation sites in the variable and C-terminal regions (Figure 11).

To date, no function has been associated with sand fly PpSP32-like proteins, although *L. longipalpis* and *P. perniciosus* proteins have been hypothesized to possess collagen binding activity [24], [25] and in *P. papatasi*, PpSP32 transcripts are expressed independently of either diet or age [87], indicating a vital role for these molecules in feeding.

**Figure 12:**
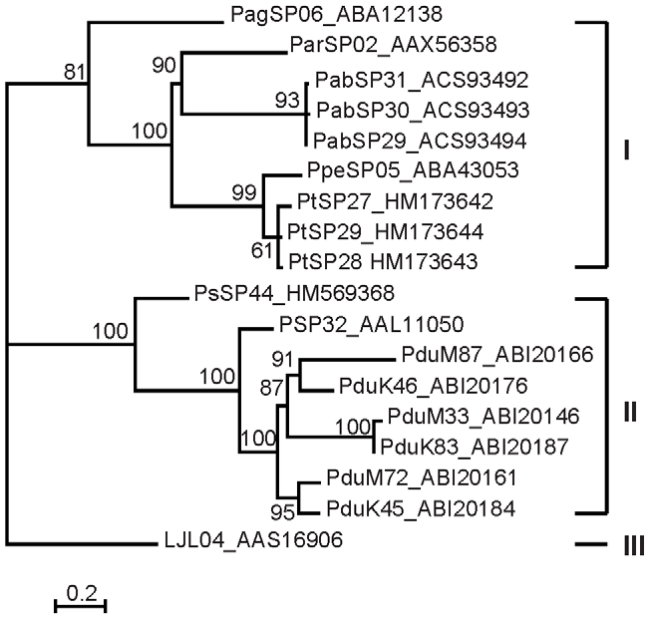
Phylogenetic analysis of the PpSP32-like family of sand fly salivary proteins. Phylogenetic analysis of PpSP32-like proteins from *Phlebotomus arabicus* (Pab), *P. argentipes* (Pag), *P. ariasi* (Par), *P. duboscqi* (Pdu), *P. papatasi* (Pp), *P. perniciosus* (Ppe), *P. sergenti* (Ps), *P. tobbi* (Pt), and *Lutzomyia longipalpis* (LJL). Phylogenetic analysis was conducted on amino acid sequences without signal peptide using Tree Puzzle (version 5.2) by maximum likelihood (WAG model), quartet puzzling, and automatically estimated internal branch node support (10,000 replications). Sequence names, accession numbers, and branch node values are indicated.

Phylogenetic analysis of PpSP32-like proteins reflects again the taxonomic relationship within Phlebotomine sand flies [33]. True to form, phylogenetic position of *P. tobbi* PpSP32-like proteins are within a subclade I with *P. perniciosus* and the *P. sergenti* PpSP32-like protein is within the *Phlebotomus* and *Paraphlebotomus* clade II (Figure 12).

### Yellow-related proteins

Phlebotomine yellow-related proteins are characterized by the presence of major royal jelly protein domain (MRJP; pfam03022). Originally, MRJP proteins were described from honeybee larval jelly, making up to 90% of the protein content [88]. Sequences related to MRJP proteins were described in *Drosophila*, where it is related to cuticle pigmentation and, when mutated, it produced a yellow phenotype and thus named Yellow proteins [89], [90]. In bloodsucking Diptera, salivary yellow-related proteins have only been described in sand flies [55], [56] and black flies [91].

**Figure 13:**
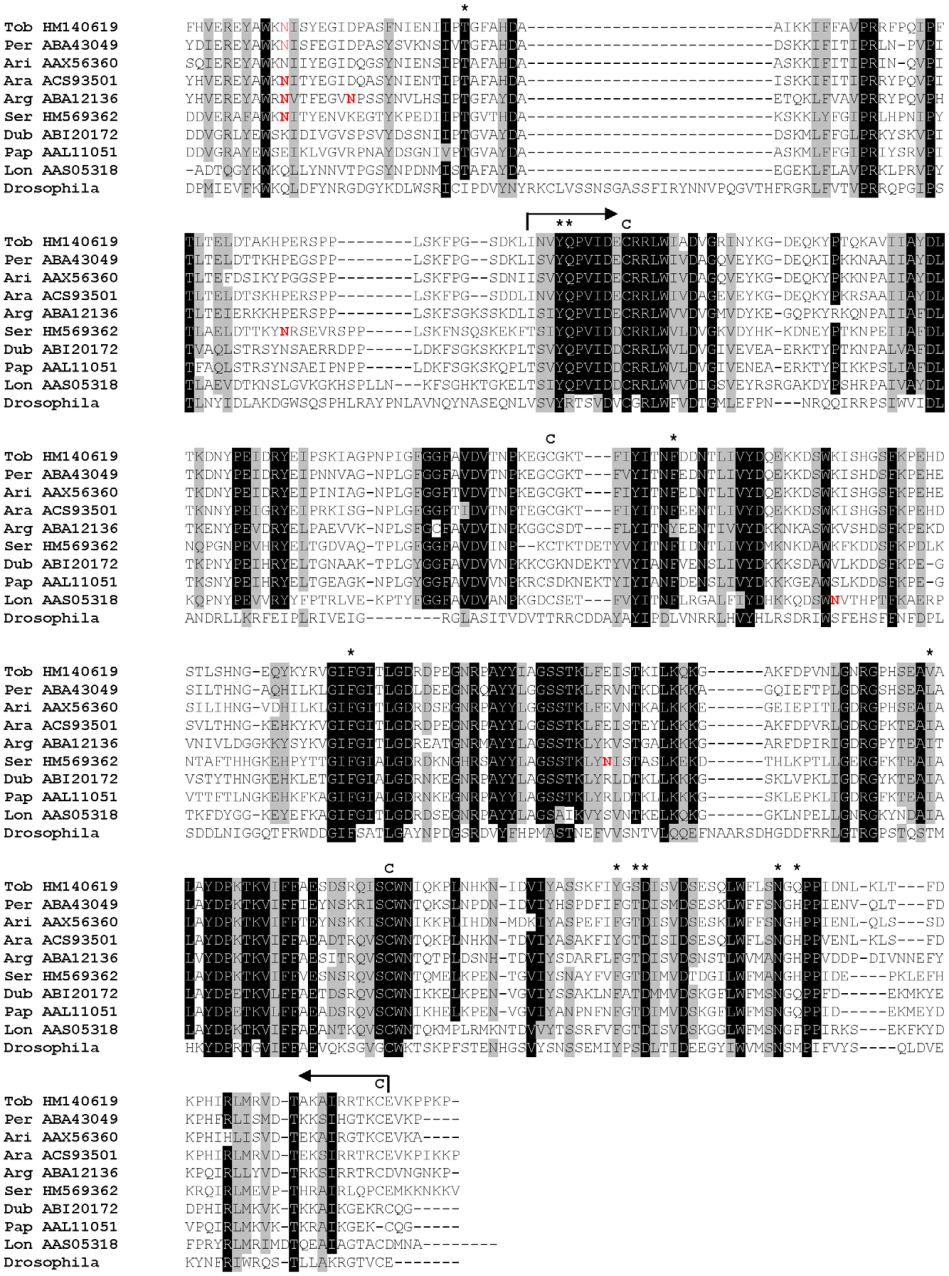
Multiple sequence alignment of the yellow-related family of salivary proteins. Multiple sequence alignment of yellow-related proteins from *Phlebotomus arabicus* (Ara), *P. argentipes* (Arg), *P. ariasi* (Ari), *P. duboscqi* (Dub), *P. papatasi* (Pap), *P. perniciosus* (Per), *P. sergenti* (Ser), *P. tobbi* (Tob), *Lutzomyia longipalpis* (Lon), and related sequence from *Drosophila simulans* (XP_002103634). Sequences without signal peptide were aligned using ClustalX and manually refined using BioEdit sequence-editing software. Accession numbers are indicated in the sequence name. Identical amino acid residues are highlighted black and similar residues grey. Red residues (N) denote predicted N-glycosylation sites. The span of the MRJP protein domain (pfam03022) is marked by arrows. Based on the crystal structure of Lon AAS05318 [9], the cystine residues forming disulfide bonds are indicated by letter C and conserved amino acids contained in the ligand binding pocket by an asterisk (*).

Yellow-related proteins are found in all sand fly species studied to date. In the *P. sergenti* cDNA library, five different clusters were found (PsSP18/HM569361, PsSP19/HM560865, PsSP20/HM560866, PsSP22/HM560867, PsSP26/HM569362) compared with *P. tobbi*, where only two clusters were found (PtSP37/HM140618 and PtSP38/HM140619) (Tables 1 and 2). Sand fly yellow-related proteins have a similar predicted molecular mass (41.5–45.2 kDa), wide range of pI (4.75–9.8), and contain four conserved cysteine residues shown to form two disulfide bonds in LJM11 (AAS05318) [9] (Table S1, Figure 13). Yellow-related proteins are modulated on a transcriptional level [87] and are likely post-translationally modified, as variants with different mobility have been detected on SDS-PAGE [6], [25] (Figure 13).

Ribeiro and Arca [55] proposed that in Phlebotomines, salivary yellow-related proteins work as kratagonists, the binders of biogenic amines. Indeed, Xu et al. [9] proved that the bacterially expressed *L. longipalpis* yellow-related proteins (LJM11, LJM17/AAD32198, and LJM111/ABB00904) bind biogenic amines, namely serotonin, catecholamines, and histamine. The proteins differed in affinity to the particular ligand, suggesting functional divergence within the family [9]. The midgut yellow protein in *Aedes aegypti* is involved in the melanization pathway as a dopachrome conversion enzyme [92]; however, in sand flies the yellow-related proteins found in the midgut lumen probably originating from swallowed saliva [93] and researchers failed to detect dopachrome convertase activity in salivary yellow-related proteins [28], [56]. In *Glossina morsitans*, the ubiquitous tissue expression of the protein suggests also a housekeeping role for yellow-related proteins [91].

Sand fly salivary yellow proteins possess antigenic properties as they are recognized by serum antibodies of experimentally bitten mice [12] and dogs [19], [21], as well as naturally exposed dogs, humans, and foxes [11], [16], [18], [21], [77], [85]. Additionally, a combination of recombinant LJM17 and LJM11 successfully substituted *L. longipalpis* whole SG sonicate in probing sera of individuals for vector exposure [16], [20].

Yellow proteins are also under consideration for anti-*Leishmania* vector-based vaccines. LJM17 from *L. longipalpis* elicited leishmanicidal Th1 cytokines in immunized dogs [8], and LJM11 protected laboratory animals against both *Le. major* and *Le. infantum* [7], [9]. In contrast, mice immunized with *P. papatasi* yellow-related proteins PpSP42 or PpSP44 (AAL11052 and AAL11051, respectively) elicited Th2 cytokines and exacerbated *Le. major* infection [84]. It remains to be elucidated whether the protection induced by yellow-related proteins is related to particular protein immunogenicity, to sand fly species, or to the vector-*Leishmania*-host combination, as all of these factors can contribute to vaccine efficacy. Recently, Xu et al. [9] showed that *L. longipalpis* LJM11 but not LJM111 produces a DTH response in mice challenged by SGH. The authors related this immunogenicity to electrostatic potential on the protein surface, which is positive in LJM11; thus the protein is probably more attractive to antigen-presenting cells [9].

**Figure 14:**
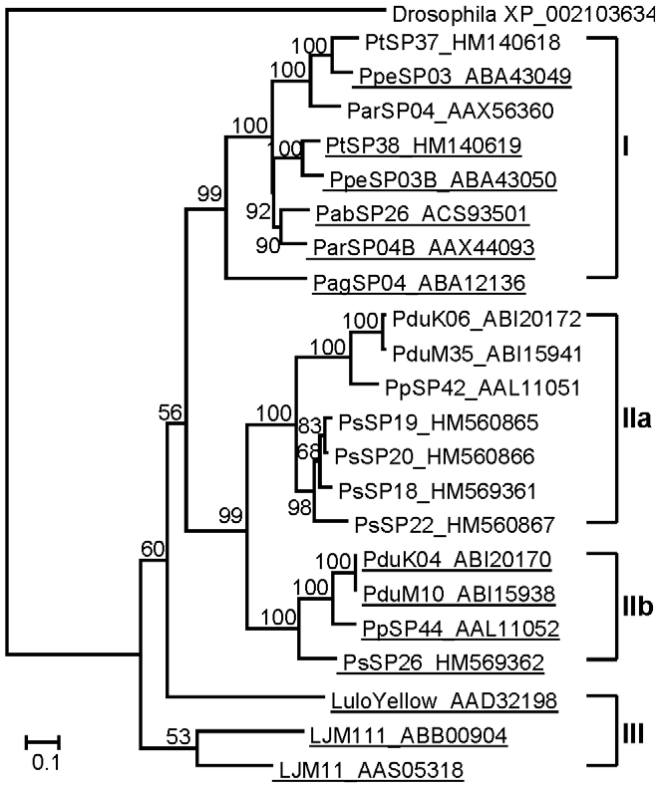
Phylogenetic analysis of the yellow-related family of sand fly salivary proteins. Phylogenetic analysis of yellow-related proteins from *Phlebotomus arabicus* (Pab), *P. argentipes* (Pag), *P. ariasi* (Par), *P. duboscqi* (Pdu), *P. papatasi* (Pp), *P. perniciosus* (Ppe), *P. sergenti* (Ps), *P. tobbi* (Pt), *Lutzomyia longipalpis* (Lulo or LJM), and related sequence from *Drosophila simulans* (XP_002103634). Phylogenetic analysis was conducted on amino acid sequences without signal peptide using Tree Puzzle (version 5.2 by) maximum likelihood (WAG model), quartet puzzling, and automatically estimated internal branch node support (10,000 replications). Sequence names, accession numbers, and branch node values are indicated. Underlined sequences possess predicted N-glycosylation sites.

Yellow-related proteins are highly conserved among sand flies. Phylogenetic analysis produced three major clades combining *Larroussius*, *Adlerius* and *Euphlebotomus* (clade I); *Phlebotomus* and *Paraphlebotomus* (clade II); and *Lutzomyia* (clade III), while subclades discerned each subgenus (Figure 14). Interestingly, *P. sergenti* illustrates a gene duplication event that preceded speciation and was followed by a clear gene duplication expansion that is seen in one of the subclades. Gene duplication in bloodsucking arthropod salivary molecules is fundamental for the functional diversification of proteins, as can be seen with the range of substrates bound by the *L. longipalpis* yellow-related proteins [9]. Within clade II, two subclades can be seen distinguishable by the presence of putative N-glycosylation sites. Moreover, sequences in clade IIa have a slightly higher predicted isoelectric point than the glycosylated sequences in clade IIb (Figure 14, Table S1), indicating another feature that might be responsible for functional diversification.

### ParSP25-like proteins

**Figure 15:**
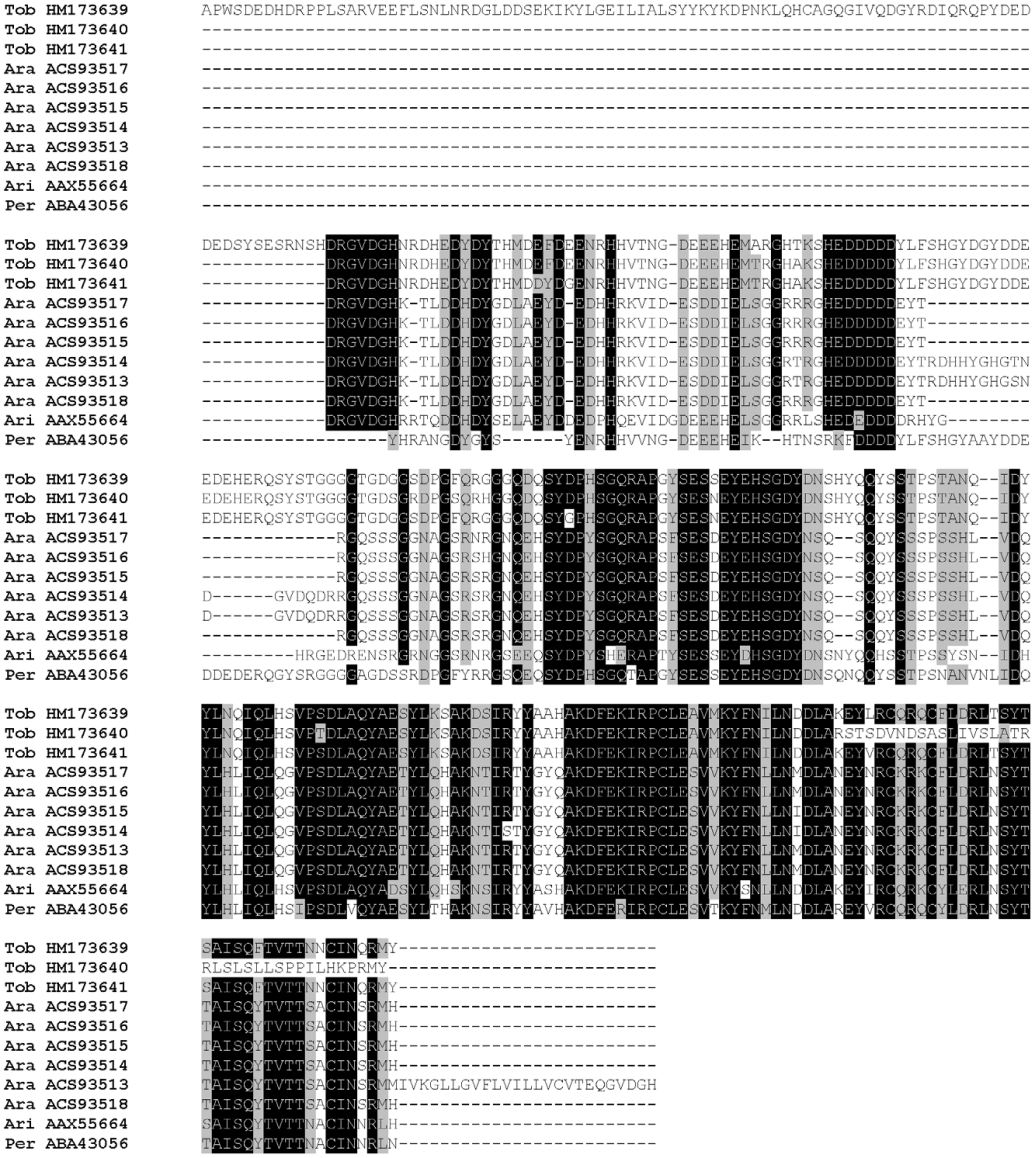
Multiple sequence alignment of the ParSP25-like family of sand fly salivary proteins. Multiple sequence alignment of *Phlebotomus tobbi* PtSP73 (HM173639), PtSP75 (HM173640), and PtSP76 (HM173641) with related sequences from *P. arabicus* (Ara), *P. ariasi* (Ari), and *P. perniciosus* (Per). Sequences without signal peptide were aligned using ClustalX and manually refined using BioEdit sequence-editing software. Accession numbers are indicated in the sequence name. Identical amino acid residues are highlighted black and similar residues grey.

ParSP25-like transcripts were found in *P. tobbi* but not in *P. sergenti* SG library. *Phlebotomus tobbi* ParSP25-like molecules (PtSP73/HM173639, PtSP75/HM173640, and PtSP76/HM173641) have predicted molecular mass ranging from 27.8 to 38.8 kDa and contain a large proportion of acidic residues resulting in a pI of 4.5±0.1. The sequences share similarity with eight other sand fly salivary proteins from three sand fly species [25], [27], [28] (Figure 15), all of them with predicted pI between 4.4 and 5.0 (Table S1). Analysis of the putative protein sequences revealed highly conserved regions rich in amino acid residues such as Asp, Tyr, Glu, and Ser and no predicted N-glycosylation sites (Figure 15). Though the function is not known, some members of this family were shown to be highly antigenic. Mice immunized with a plasmid coding for ParSP25 (AAX55664) elicited high levels of anti-*P. ariasi* IgG1 and a strong DTH reaction when challenged with *P. ariasi* saliva [27]. Moreover, dogs exposed to *P. perniciosus* bites strongly bind to protein band characterized as PpeSP08 (ABA43056) [21]. Sand fly ParSP25-like proteins are most likely genus-specific because, so far, the sequences have been found only in *Adlerius* (*P. arabicus*) and *Larroussius* species (*P. ariasi*, *P. perniciosus*, *P. tobbi*) and not in representatives of the other subgenera (Figure 15).

### The 33-kDa family

**Figure 16:**
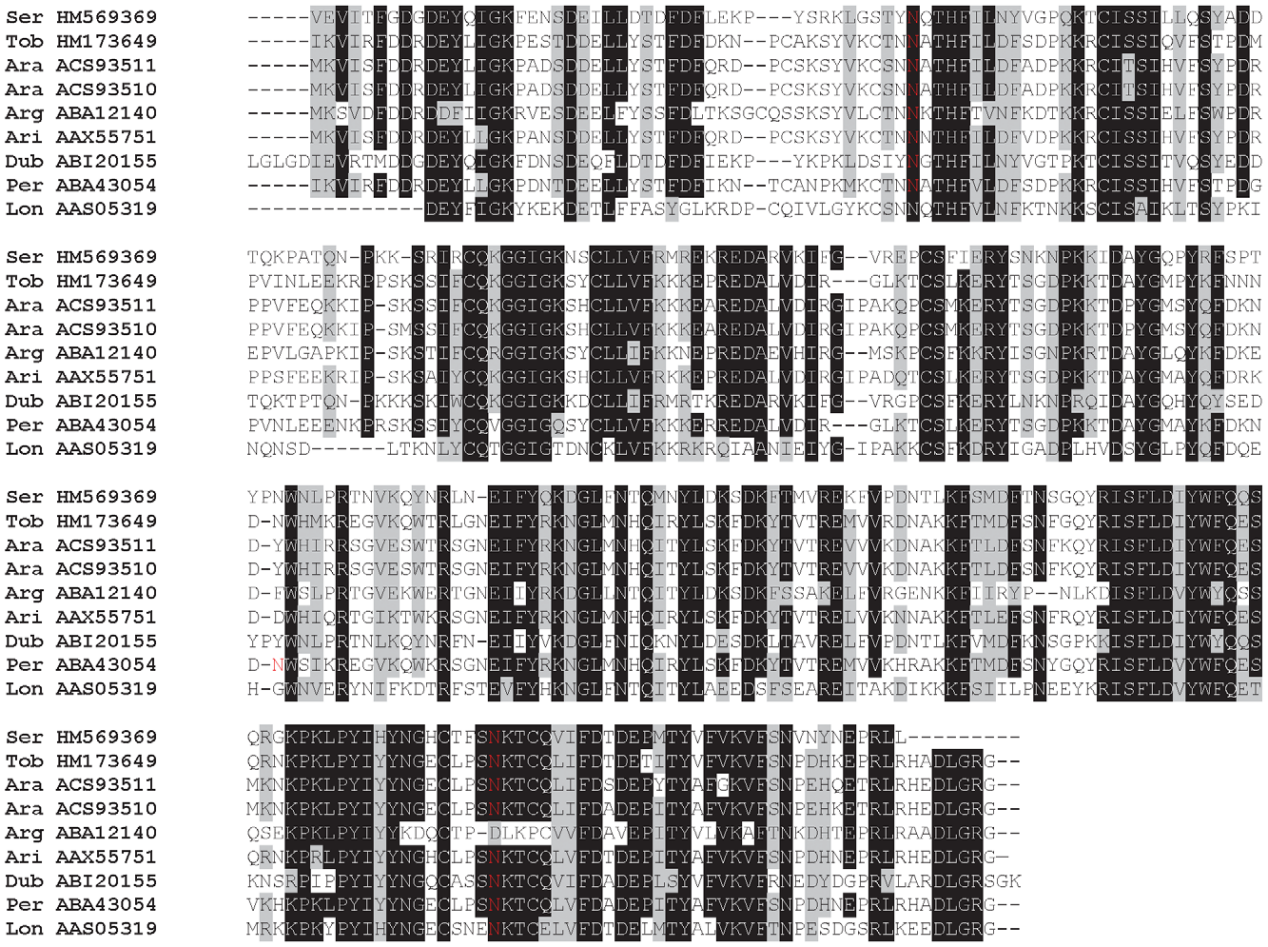
Multiple sequence alignment of the 33-kDa salivary protein family. Multiple sequence alignment of *Phlebotomus sergenti* PsSP49 (HM569369) and P. *tobbi* PtSP66 (HM173645) proteins with related sequences from *P. arabicus* (Ara), *P. argentipes* (Arg), *P. ariasi* (Ari), *P. duboscqi* (Dub), *P. perniciosus* (Per), and *Lutzomyia longipalpis* (Lon). Sequences without signal peptide were aligned using ClustalX and manually refined using BioEdit sequence-editing software. Accession numbers are indicated in the sequence name. Identical amino acid residues are highlighted black and similar residues grey. Red residues (N) denote predicted N-glycosylation sites.

These proteins, named by Anderson et al. [25] as members of the 33-kDa family, have not yet been found in any Diptera other than sand flies. PsSP49 (HM569369) and PtSP66 (HM173645) share sequence similarity with seven other sand fly salivary proteins from six sand fly species both from both New and Old World sand flies [24]–[28] (Figure 16). All sand fly 33-kDa family proteins have similar predicted molecular weight (32.3–34.5 kDa) and alkalic pI (8.2–9.1) (Table S1). PsSP49 and PtSP66 were both identified in the proteomic analysis (Figure 1). Two highly conserved N-glycosylation sites were predicted among all sand fly sequences (Figure 16) and both PsSP49 and PtSP66 were found above the predicted molecular weight in the proteomic analysis (Figure 1, Tables 1 and 2), indicating a post-translational modification. Indeed, the two proteins from *P. arabicus* (PabSP32/ACS93510 and PabSP34/ACS93511) showed glycosylation by ProQ Emerald staining [28]. The function is unknown; however, *P. perniciosus* PpeSP06 (ABA43054) and the *L. longipalpis* LJL143 (AAS05319) were identified as antigens for dogs living in endemic areas of *Le. infantum* [16], [21], the later one also shown to be a candidate for vaccine against canine leishmaniasis [8].

### 41.9-kDa superfamily

**Figure 17:**
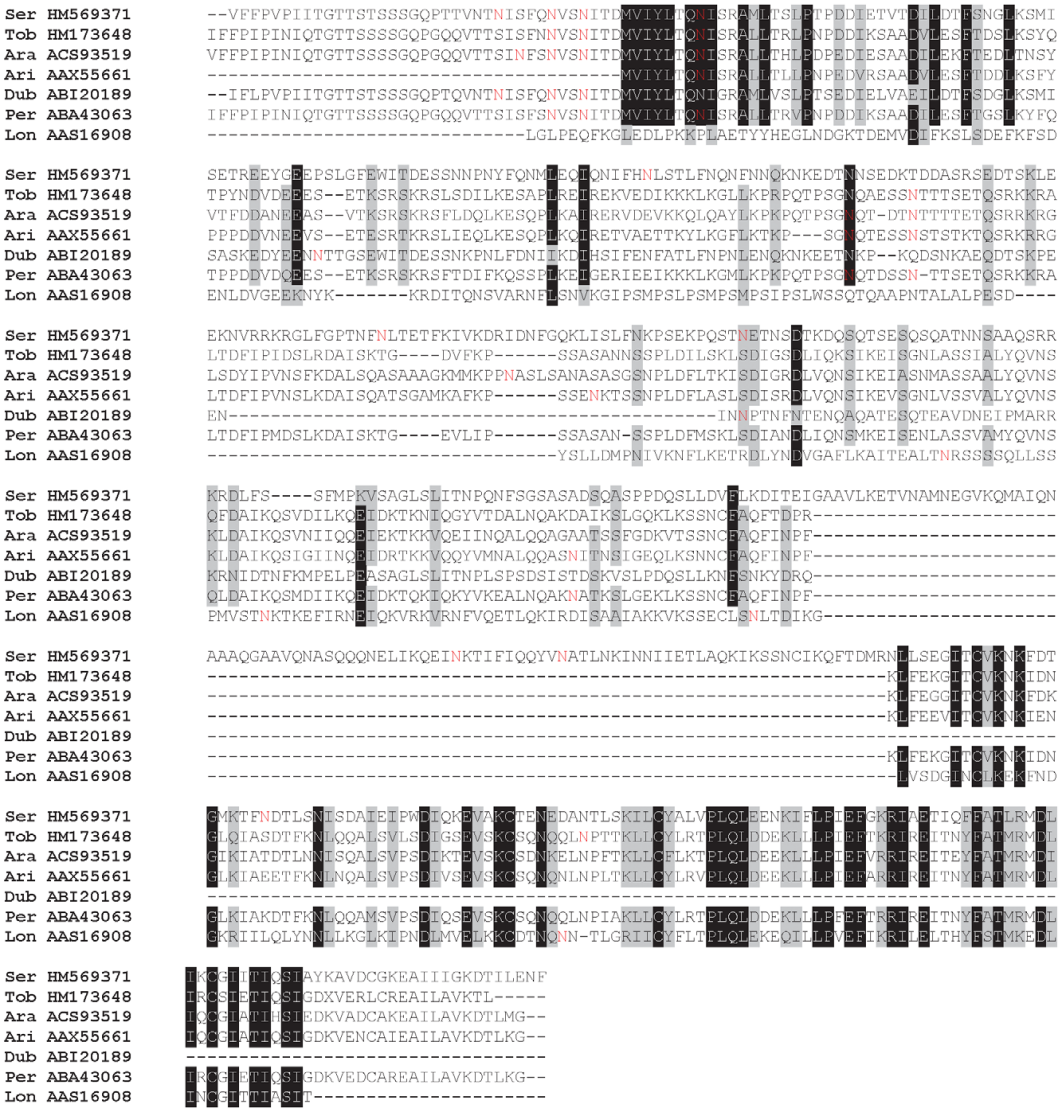
Multiple sequence alignment of the sand fly members of 41.9-kDa salivary protein superfamily. Multiple sequence alignment of *Phlebotomus sergenti* PsSP82 (HM569371) and P. *tobbi* PtSP49 (HM173648) proteins with related sequences from *P. arabicus* (Ara), *P. ariasi* (Ari), *P. duboscqi* (Dub), *P. perniciosus* (Per), and *Lutzomyia longipalpis* (Lon). Sequences without signal peptide were aligned using ClustalX and manually refined using BioEdit sequence-editing software. Accession numbers are indicated in the sequence name. Identical amino acid residues are highlighted black and similar residues grey. Red residues (N) denote predicted N-glycosylation sites.

41.9-kDa protein superfamily is specific to bloodsucking Nematocera encompassing members of mosquitoes, biting midges, black flies, and sand flies [56]. The *P. sergenti* and *P. tobbi* members of this superfamily, PsSP82 (HM569371) and PtSP49 (HM173648), share sequence similarity with five other sand fly salivary proteins from five sand fly species (Figure 17). These sand fly proteins have a wide range of predicted molecular weight (27.5–56.6 kDa) and pI (4.3–8.5) (Table S1) but only one of them, *P. perniciosus* PpeSP19 (ABA43063), has been found by proteomic analysis [25]. All sequences are rich in putative N-glycosylation sites (Figure 17) and the function is not known.

### Other putative salivary proteins

Several other putative salivary proteins were identified in the transcriptomes of *P. tobbi* and *P. sergenti* SGs. They are smaller than 15 kDa, their function is not known, and are, thus far, unique to sand flies. Additionally, none of these small proteins have been found in the proteomic analysis (Figure 1, Tables 1 and 2).

**Figure 18:**
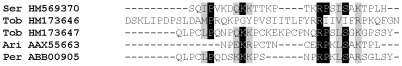
Multiple sequence alignment of the PsSP28, PtSP8, and PtSP81 salivary proteins. Multiple sequence alignment of *Phlebotomus sergenti* PsSP28 (HM569370) and P. *tobbi* PtSP8 (HM173646) and PtSP81 (HM173647) proteins with related sequences from *P. ariasi* (Ari) and *P. perniciosus* (Per). Sequences without signal peptide were aligned using ClustalX and manually refined using BioEdit sequence-editing software. Accession numbers are indicated in the sequence name. Identical amino acid residues are highlighted black and similar residues grey.

PsSP28 (HM569370), PtSP8 (HM173646), and PtSP81 (HM173647) share sequence similarity with *P. ariasi* ParSP23 (AAX55663) and *P. perniciosus* PpeSP15 (ABB00905) (Figure 18). The proteins have a low predicted molecular weight (2.4–5.0 kDa) and an alkalic pI (9.2–10.7).

**Figure 19:**
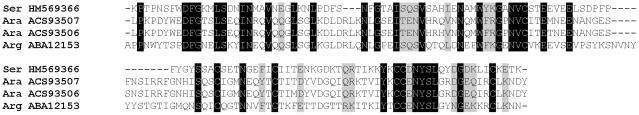
Multiple sequence alignment of the *Phlebotomus sergenti* PsSP98 salivary protein. Multiple sequence alignment of *Phlebotomus sergenti* PsSP98 protein (HM569366) with related sequences from *P. arabicus* (Ara) and *P. argentipes* (Arg). Sequences without signal peptide were aligned using ClustalX and manually refined using BioEdit sequence-editing software. Accession numbers are indicated in the sequence name. Identical amino acid residues are highlighted black and similar residues grey.

PsSP98 (HM569366) has a predicted molecular weight similar to PpSP15-like proteins (14.3 kDa) but is highly acidic (pI = 4.73). The protein sequence is related to 16-kDa proteins from *P. arabicus* (PabSP64/ACS93507, PabSP63/ACS93506) and *P. argentipes* (PagSP73/ABA12153) (Figure 19).

**Figure 20:**
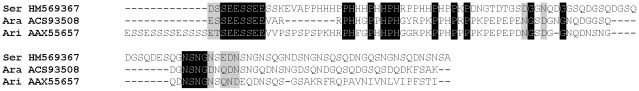
Multiple sequence alignment of the *Phlebotomus sergenti* PsSP73 salivary protein. Multiple sequence alignment of *Phlebotomus sergenti* PsSP73 protein (HM569367) with related sequences from *P. arabicus* (Ara) and *P. ariasi* (Ari). Sequences without signal peptide were aligned using ClustalX and manually refined using BioEdit sequence-editing software. Accession numbers are indicated in the sequence name. Identical amino acid residues are highlighted black and similar residues grey.

PsSP73 (HM569367) has a predicted molecular weight 12.2 kDa and is highly acidic (pI = 4.51). The predicted protein sequence is related to proteins found in *P. arabicus* (PabSP75/ACS93508) and *P. ariasi* (ParSP13/AAX55657) (Figure 20).

**Figure 21:**
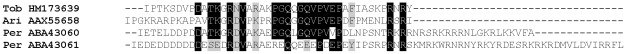
Multiple sequence alignment of the *Phlebotomus tobbi* PtSP71 salivary protein. Multiple sequence alignment of *Phlebotomus tobbi* PtSP73 protein (HM173639) with related sequences from *P. ariasi* (Ari) and *P. perniciosus* (Per). Sequences without signal peptide were aligned using ClustalX and manually refined using BioEdit sequence-editing software. Accession numbers are indicated in the sequence name. Identical amino acid residues are highlighted black and similar residues grey.

PtSP71 (HM173638) has a low predicted molecular weight (4.5 kDa) and an alkalic pI (10.6). The protein sequence is related to molecules identified in *P. perniciosus* (PpeSP12/ABA43060, PpeSP13/ABA43061) and *P. ariasi* (ParSP15/AAX55658) (Figure 21), indicating these sequences might be unique to *Larroussius* species.

### Antigens and glycoproteins of *Phlebotomus tobbi* salivary proteins

**Figure 22:**
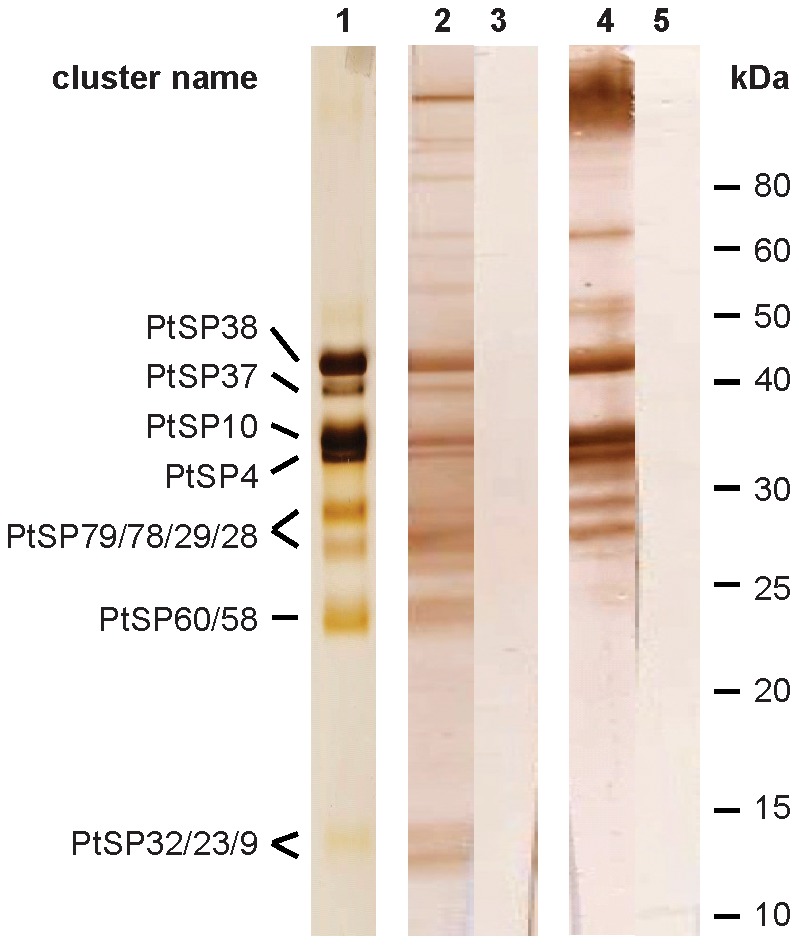
*Phlebotomus tobbi* salivary gland antigens and glycoproteins. Salivary gland homogenate of *Phlebotomus tobbi* was separated on 8% polyacrylamide gel under non-reducing conditions. Separated proteins were silver stained (lane 1) or electrotransferred to nitrocellulose membrane and incubated with (2) serum from rabbit repeatedly exposed to bites of *P. tobbi* females, (3) non-immune rabbit serum, (4) Concanavalin A lectin, or (5) ConA preincubated with methyl-α-D-mannopyranoside to control specificity of the ConA reaction. Probable cluster name as compared to mass spectrometry results in Figure 1 and molecular weight (kDa) are indicated on the left and right side, respectively.

To identify antigens and glycoproteins in *P. tobbi* SGH, electrophoretically separated proteins were incubated with anti-*P. tobbi* rabbit serum and a lectin Concanavalin A (ConA), respectively (Figure 22). When compared with the proteome analysis in Figure 1, the protein bands visible by silver staining are most likely yellow-related proteins (PtSP37 and PtSP38), apyrases (PtSP4 and PtSP10), antigen 5-related proteins (PtSP77 and PtSP79), PpSP32-like proteins (PtSP28 and PtSP29), D7-related proteins (PtSP58 and PtSP60), and PpSP15-like proteins (PtSP9, PtSP23, and PtSP32). Anti-*P. tobbi* antibodies recognized all identified bands as well as other six high molecular weight proteins not visible by silver staining (Figure 22, lane 2).

Most of the *P. tobbi* proteins reacted with ConA, indicating they are N-glycosylated. The lectin binding was specific, as the reactivity was totally inhibited when ConA was preincubated with specific monosaccharide methyl-α-D-mannopyranoside. The most intense reaction was observed with the high molecular weight band not visible by silver staining, and with the bands of molecular weight similar to one yellow-related protein (PtSP38) and both apyrases. Among the nine silver-stained bands, three did not react with ConA, namely bands with molecular weight similar to D7-related proteins, PpSP15-like proteins, and one yellow-related protein (PtSP37) (Figure 22, lane 4). The reactivity with ConA is in agreement with N-glycosylation as predicted by NetNGlyc server [48], with the exception of PtSP10 apyrase (Table 3).

**Table 3:** N-glycosylation sites of *Phlebotomus tobbi* salivary proteins.

Cluster name	GenBank Accn	Comment	kDa	Position	Potential score	Jury agreement	N-Glyc result	ConA result
PtSP38	HM140619	yellow-related	41.7	29 NISY	0.6242	8/9	+	+
PtSP37	HM140618	yellow-related	39.2	No sites predicted in this sequence				−
PtSP10	HM135952	apyrase	37.6	No sites predicted in this sequence				+
PtSP4	HM135951	apyrase	33.0	163 NISK	0.6696	9/9	++	+
				248 NFSD	0.5678	8/9	+	+
PtSP79	HM140622	antigen 5-related	29.6	160 NITR	0.6879	9/9	++	+
PtSP77	HM140620	antigen 5-related	29.6	159 NITR	0.6931	9/9	++	+
PtSP29	HM173644	PpSP32-like	29.6	111 NPTG*	0.6866	9/9	++	+
PtSP28	HM173643	PpSP32-like	29.6	111 NPTG*	0.6868	9/9	++	+
PtSP60	HM164150	D7-related	24.5	No sites predicted in this sequence				−
PtSP58	HM164149	D7-related	24.5	No sites predicted in this sequence				−
PtSP32	HM164144	PpSP15-like	13.5	No sites predicted in this sequence				−
PtSP23	HM164142	PpSP15-like	13.5	No sites predicted in this sequence				−
PtSP9	HM164139	PpSP15-like	13.5	No sites predicted in this sequence				−

Selected mature proteins from *Phlebotomus tobbi* were subjected to the NetNGlyc 1.0 Server [48] using the default setting (by default, predictions are done only on the Asn-Xaa-Ser/Thr sequons and the treshold is set up at 0.5). Cluster names and GenBank accession numbers are indicated. Molecular weight (kDa) was calculated based on the Figure 1A. The Position column defines predicted glycosylated sites. The Potential score is the averaged output of nine neural network, and the Jury agreement column indicates how many of the nine networks support the prediction. The N-Glyc Result column shows putative glycosylated sites: + denotes Potential >0.5, ++ Potential >0.5 and Jury agreement (9/9) or Potential >0.75, +++ Potential >0.75 and Jury agreement, ++++ Potential >0.90 and Jury agreement.

*: Proline occurs just after the asparagine residue that makes it highly unlikely that the asparagine is glycosylated, presumably due to conformational constraints. ConA column shows reactivity with lectin concanavalin A based on the Figure 22, lane 4.

We can speculate that the most glycosylated band with the highest molecular weight might be hyaluronidase. Although producing a minor unstainable band, it is predicted to be highly glycosylated (Figure 7) and its activity is clearly visible around 135 kDa in zymography analyses (Figure 6).

Within sand fly yellow-related proteins, it is common that glycosylated and non-glycosylated forms occur in the same species. As proved for *P. papatasi* [93] and predicted for protein sequences of *Phlebotomus* (*P. papatasi* and *P. duboscqi*) and *Paraphlebotomus* (*P. sergenti*) species, at least one form is glycosylated, forming a well supported subclade with glycosylated sequences from other species (Figure 14). Glycosylated and non-glycosylated forms are also present in *P. tobbi*, as proven by blot analysis (Figure 22), although the closely related *P. perniciosus* possesses only glycosylated forms. Interestingly, in sand fly species within the clades I and III (Figure 14), all published sequences are glycosylated with an exception of *P. tobbi* and *P. ariasi*, which has at least one non-glycosylated form. Further research is needed to investigate whether the presence of sugar side chains may contribute to the antigenicity of the yellow-related proteins.

### Conclusions

With over 80 species of sand flies implicated in *Leishmania* transmission, it is vital to continue describing their salivary proteins in the search for vaccine candidates and markers of exposure. In this study, we prepared and analyzed the transcriptome and proteome data of *P. tobbi* and *P. sergenti* to broaden our knowledge on the repertoire of *Larroussius* salivary proteins and provide the first report from a *Paraphlebotomus* sand fly, respectively.

*P. tobbi* has been reported to transmit *Le. infantum* that causes cutaneous leishmaniasis [32]. Interestingly, the salivary proteins of *P. tobbi* are highly homologous to those of *P. perniciosus*, a vector of *Le. infantum* that causes visceral disease. It is likely that, in this instance, the salivary proteins of *P. tobbi* are not the determining factor for these different disease manifestations. However, in general, it is possible that the divergence, diversity or amount of sand fly salivary proteins or non proteinaceous components of the saliva correlate with different disease manifestations of the same species of *Leishmania*.

The transcriptome data can be utilized to prepare recombinant proteins that can be used to test their potential as anti-*Leishmania* vaccines or in epidemiologic studies to develop more specific and efficient methods for measurement of vector exposure. Finally, recombinant salivary proteins may also help us to understand the mechanism of blood sucking or find biological activities of many of these novel sequences.

## Supporting Information

Figure S1 ***Phlebotomus tobbi*** **and** ***P. sergenti*** **protein families.** Analysis of salivary proteins from *Phlebotomus tobbi* (Pt) and *P. sergenti* (Ps). Phylogenetic analysis was conducted on amino acid sequences with signal peptide using Tree Puzzle (version 5.2) by maximum likelihood (WAG model), quartet puzzling, and automatically estimated internal branch node support (10,000 replications). Sequence cluster names and branch node values are indicated. Protein families are listed on the right.
(TIFF)

Figure S2 **Multiple sequence alignment of the PpSP15-like family of salivary proteins.** Multiple sequence alignment of the PpSP15-like proteins from *Phlebotomus arabicus* (Ara), *P. argentipes* (Arg), *P. ariasi* (Ari), *P. duboscqi* (Dub), *P. papatasi* (Pap), *P. perniciosus* (Per), *P. sergenti* (Ser), *P. tobbi* (Tob), and *Lutzomyia longipalpis* (Lon). Sequences without signal peptide were aligned using ClustalX and manually refined using BioEdit sequence-editing software. Accession numbers are indicated in the sequence name. Identical amino acid residues are highlighted black and similar residues grey.
(TIFF)

Table S1 **List of sand fly salivary proteins with their identifiers and selected protein features.** Published sand fly salivary proteins from *Phlebotomus arabicus* (Ara), *P. argentipes* (Arg), *P. ariasi* (Ari), *P. duboscqi* (Dub), *P. papatasi* (Pap), *P. perniciosus* (Per), *P. sergenti* (Ser), *P. tobbi* (Tob), and *Lutzomyia longipalpis* (Lon). The proteins are listed with their house name, GenBank accession numbers (Accn) for both nucleotide and protein sequences, protein name, presence in the proteome analysis as confirmed by mass spectrometry or Edman degradation, protein family, predicted signal peptide (SignalP), putative mature protein features (pI, predicted isoelectric point; Mw, predicted molecular weight; AA, number of amino acid residues), and reference.
(XLS)

Text S1 **Accession numbers for genes and proteins mentioned in the text including tables, figure and supplemental files.**
(DOC)
