# Correction: Salivary Gland Transcriptomes and Proteomes of *Phlebotomus tobbi* and *Phlebotomus sergenti*, Vectors of Leishmaniasis

**DOI:** 10.1371/annotation/fb586825-2ad7-41a5-b7d6-aef0e65c5887

**Published:** 2012-06-22

**Authors:** Iva Rohoušová, Sreenath Subrahmanyam, Věra Volfová, Jianbing Mu, Petr Volf, Jesus G. Valenzuela, Ryan C. Jochim

The following information was missing from the funding statement: This project was partially funded by EU grant 2011-261504 EDENEXT and the paper is cataloged by the EDENEXT Steering Committee as EDENEXT034. 

