# Supplementary figures and images for: Salivary Gland Transcriptomes and Proteomes of Phlebotomus tobbi and Phlebotomus sergenti, Vectors of Leishmaniasis
Source: PLoS Negl Trop Dis. 2012 May 22;6(5):e1660. doi: 10.1371/journal.pntd.0001660 (PMC3358328; doi:10.1371/journal.pntd.0001660)

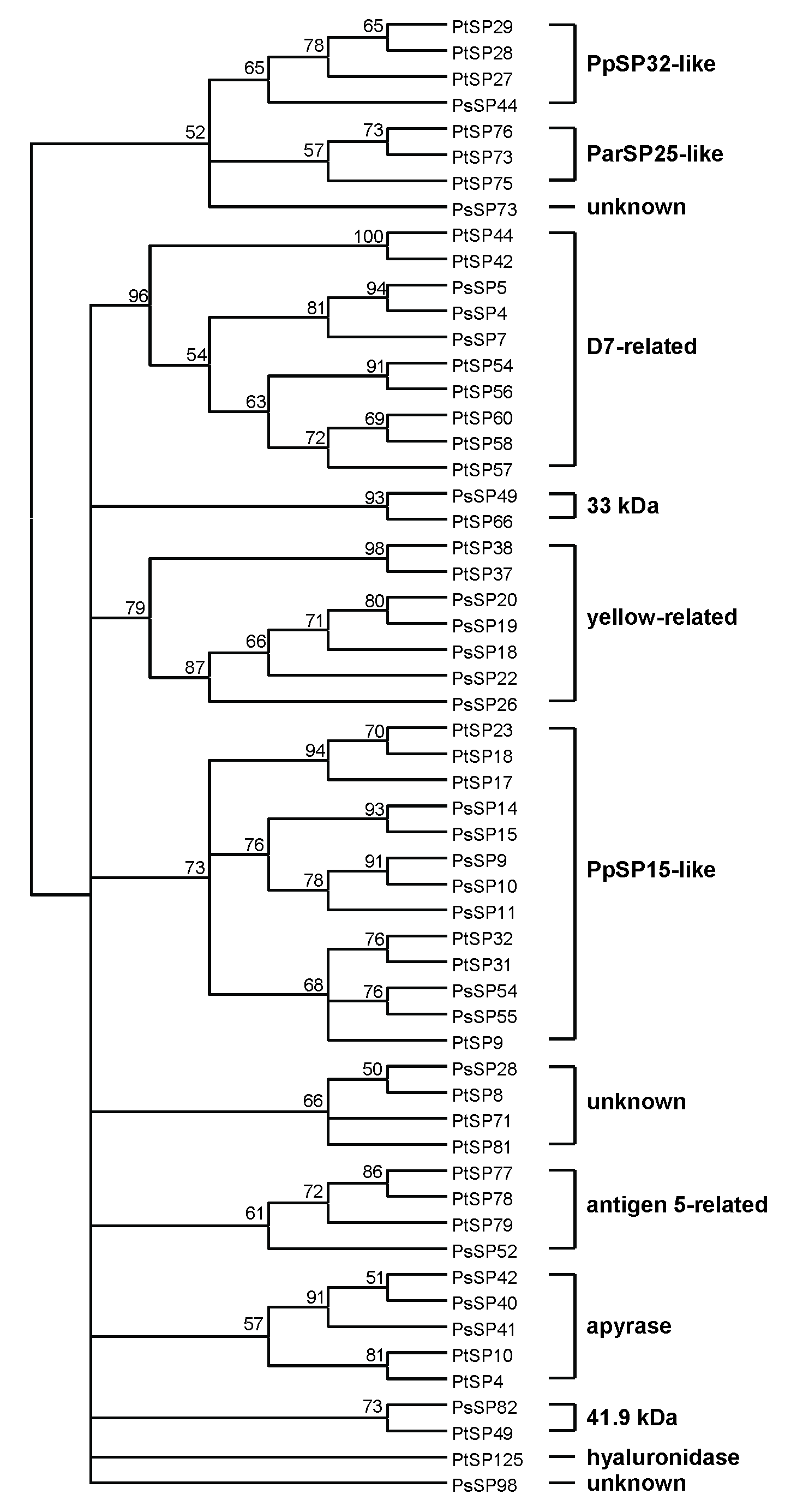

Supplement: Figure S1 — Phlebotomus tobbi and P. sergenti protein families. Analysis of salivary proteins from Phlebotomus tobbi (Pt) and P. sergenti (Ps). Phylogenetic analysis was conducted on amino acid sequences with signal peptide using Tree Puzzle (version 5.2) by maximum likelihood (WAG model), quartet puzzling, and automatically estimated internal branch node support (10,000 replications). Sequence cluster names and branch node values are indicated. Protein families are listed on the right. (TIFF) [file pntd.0001660.s001.tif]

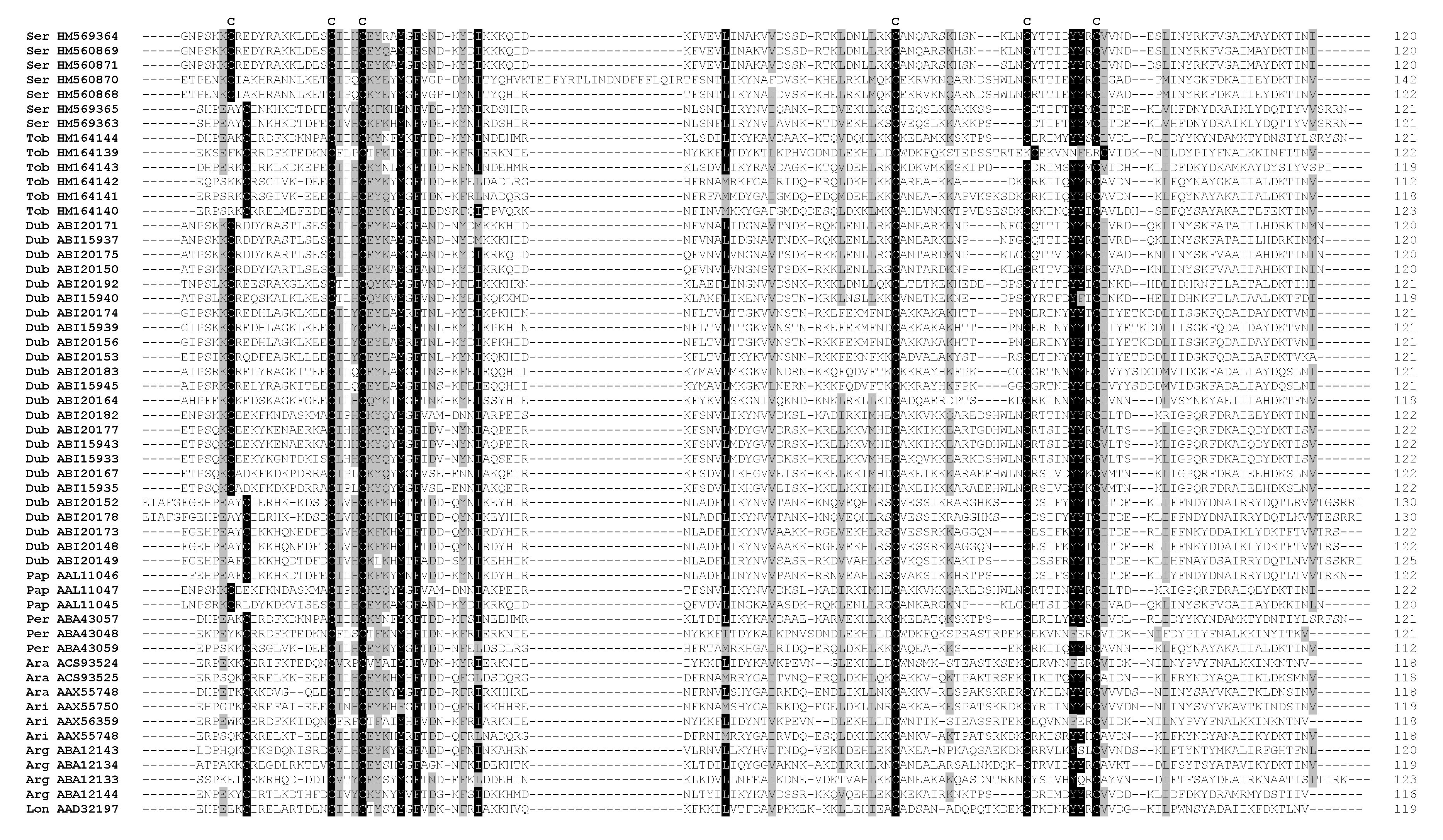

Supplement: Figure S2 — Multiple sequence alignment of the PpSP15-like family of salivary proteins. Multiple sequence alignment of the PpSP15-like proteins from Phlebotomus arabicus (Ara), P. argentipes (Arg), P. ariasi (Ari), P. duboscqi (Dub), P. papatasi (Pap), P. perniciosus (Per), P. sergenti (Ser), P. tobbi (Tob), and Lutzomyia longipalpis (Lon). Sequences without signal peptide were aligned using ClustalX and manually refined using BioEdit sequence-editing software. Accession numbers are indicated in the sequence name. Identical amino acid residues are highlighted black and similar residues grey. (TIFF) [file pntd.0001660.s002.tif]
