# Supplementary material for: Salivary Gland Transcriptomes and Proteomes of Phlebotomus tobbi and Phlebotomus sergenti, Vectors of Leishmaniasis
Source: PLoS Negl Trop Dis. 2012 May 22;6(5):e1660. doi: 10.1371/journal.pntd.0001660 (PMC3358328; doi:10.1371/journal.pntd.0001660)
Supplement: Text S1 — Accession numbers for genes and proteins mentioned in the text including tables, figure and supplemental files. (DOC) [file pntd.0001660.s004.doc]

**Comparative analysis of salivary gland transcriptomics with respect to vectors of cutaneous and visceral leishmaniasis**

**Iva Rohoušová^1^, Sreenath Subrahmanyam^2^, Věra Volfová^1^, Jianbing Mu^3^, Petr Volf ^1^, Jesus G. Valenzuela^2,*^, Ryan C. Jochim^2^,***

**1** Charles University in Prague, Faculty of Science, Department of Parasitology, Prague, Czech Republic, **2** Vector Molecular Biology Unit, Laboratory of Malaria and Vector Research, and **3** Malaria Genomics Section, National Institute of Allergy and Infectious Diseases, National Institutes of Health, Rockville, Maryland, USA

* E-mail: jvalenzuela@niaid.nih.gov, rjochim@niaid.nih.gov

**Accession numbers for genes and proteins mentioned in the text including tables, figure and supplemental files**

1FCU; 2QEV; AAA29288; AAD09177; AAD32190 - AAD32192; AAD32195 - AAD32198; AAD33512; AAD33513; AAF78901; AAG17637; AAL11045 - AAL11052; AAL16051; AAR99723 - AAR99725; AAS05317 - AAS05319; AAS16906 - AAS16919; AAS17936; AAS17937; AAX44092; AAX44093; AAX54852; AAX54853; AAX55657; AAX55658; AAX55660 - AAX55668; AAX55748 - AAX55752; AAX56357 - AAX56360; ABA12133 - ABA12155; ABA39525; ABA39526; ABA43048 - ABA43064; ABA54266; ABB00902 - ABB00907; ABI15933 - ABI15945; ABI20146 - ABI20193; ACH56843; ACH56844; ACH56930; ACM40909; ACS93489 - ACS93526; ADJ54077- ADJ54132; ADJ67266; ADM18346; AF131932; AF131933; AF132510 - AF132512; AF132515 - AF132518; AF234182; AF261768; AF335485 - AF335492; AF420274; AY438269 - AY438271; AY445934 - AY445936; AY452695; AY453401; AY455906 - AY455919; AY845193 - AY845196; AY850691; AY850692; AY861654 - AY861658; AY861671; AY861672; AY862484; AY862485; AY862991 - AY862999; BAF93867; DQ136148 - DQ136170; DQ150620 - DQ150624; DQ153099 - DQ153107; DQ154097 - DQ154099; DQ190946; DQ190947; DQ192486 - DQ192491; DQ205724; DQ826514 - DQ826526; DQ834330 - DQ834343; DQ835355 - DQ835388; EZ000625 - EZ000636; FJ410293; FJ427208; FJ439531 - FJ439533; FJ474085 - FJ474088; FJ489241; FJ489242; FJ538099 - FJ538113; GW813185 - GW814274; GW814275 - GW815416; HM135951; HM135952; HM140618 - HM140622; HM164139 - HM164151; HM173638 - HM173648; HM537134; HM560860 - HM560871; HM569360 - HM569371; JN192442; M77090; NP_036401; P30659; Q8WVQ1; Q9XZ43; XP_002061131; XP_002103634; XP_551869
